# Reactivity of Cyanobacteria Metabolites with Ozone: Multicompound Competition Kinetics

**DOI:** 10.1021/acs.est.4c02242

**Published:** 2024-06-17

**Authors:** Valentin Rougé, Urs von Gunten, Elisabeth M.L. Janssen

**Affiliations:** † Eawag, Swiss Federal Institute of Aquatic Science and Technology, 8600 Dübendorf, Switzerland; ‡ School of Architecture, Civil and Environmental Engineering (ENAC), École Polytechnique Fédérale de Lausanne (EPFL), 1015 Lausanne, Switzerland

**Keywords:** cyanopeptides, planktothrix, microcystis, micropollutant, ozonation, toxins, microcystin

## Abstract

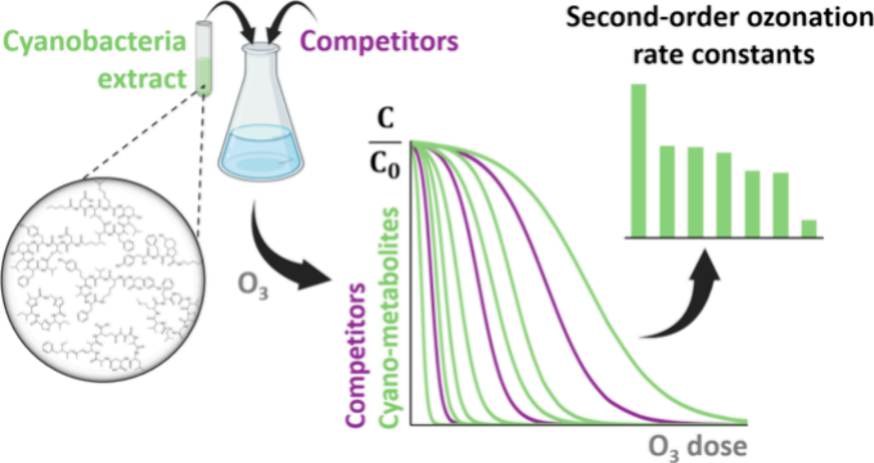

Cyanobacterial blooms occur at increasing frequency and intensity, notably in freshwater. This leads to the introduction of complex mixtures of their products, i.e., cyano-metabolites, to drinking water treatment plants. To assess the fate of cyano-metabolite mixtures during ozonation, a novel multicompound ozone (O_3_) competition kinetics method was developed. Sixteen competitors with known second-order rate constants for their reaction with O_3_ ranging between 1 and 10^8^ M^–1^ s^–1^ were applied to cover a wide range of the O_3_ reactivity. The apparent second-order rate constants (*k*_app,O3_) at pH 7 were simultaneously determined for 31 cyano-metabolites. *k*_app,O3_ for olefin- and phenol-containing cyano-metabolites were consistent with their expected reactivity (0.4–1.7 × 10^6^ M^–1^ s^–1^) while *k*_app,O3_ for tryptophan- and thioether-containing cyano-metabolites were significantly higher than expected (3.4–7.3 × 10^7^ M^–1^ s^–1^). Cyano-metabolites containing these moieties are predicted to be well abated during ozonation. For cyano-metabolites containing heterocycles, *k*_app,O3_ varied from <10^2^ to 5.0 × 10^3^ M^–1^ s^–1^, giving first insights into the O_3_ reactivity of this class of compounds. Due to lower O_3_ reactivities, heterocycle- and aliphatic amine-containing cyano-metabolites may be only partially degraded by a direct O_3_ reaction near circumneutral pH. Hydroxyl radicals, which are formed during ozonation, may be more important for their abatement. This novel multicompound kinetic method allows a high-throughput screening of ozonation kinetics.

## Abstract 2

Cyanobacteria metabolites are becoming an increasing concern for water supply. This study reports second-order rate constants for the reactions of 31 cyanobacteria metabolites with ozone determined simultaneously by a novel multicompound competition kinetic method.

## Introduction

Cyanobacteria are among the most ubiquitous organisms on the globe, comprising almost 2000 identified species living in freshwater, terrestrial, or marine environments.^1,2^ Some cyanobacteria can form dense blooms that can cause significant deterioration of the water quality, notably by increasing turbidity, depleting oxygen by decomposition of biomass after blooms subside, and releasing toxins.^3^ These blooms can occur in freshwater reservoirs that are resources for drinking water treatment plants and have been increasing in intensity and frequency worldwide in the last decades.^3,4^ Toxins can be part of a complex mixture of metabolites produced by cyanobacteria (i.e., cyano-metabolites) and can enter water treatment plants.^5^ If no appropriate treatment is in place, toxins may even end up in the finished drinking water, requiring temporary safety warnings to the population.^6,7^ Recognizing the human health concerns, the World Health Organization proposes chronic, lifetime, and acute short-term drinking water guideline values for cyanobacterial toxins.^8^ Therefore, water suppliers need to account for the presence of cyano-metabolites in raw water, anticipate the probable intensification of blooming events, and develop mitigation strategies at the source and/or appropriate treatment.

Ozone (O_3_) is a well-established and widely applied oxidant for disinfection, micropollutant degradation, and mitigation of disinfection byproducts, which has many advantages over other chemical oxidants.^9,10,15^ It can be used to efficiently degrade known potent toxins such as microcystins.^11,12^ However, a large number and diversity of other cyano-metabolites exist, with 2425 currently known compounds listed in a shared database for cyano-metabolites (CyanoMetDB, version 02, 2023), for which ozonation kinetics have not been studied to date.^13,14^ Therefore, further investigations of the reactivity of O_3_ with cyano-metabolites are needed to better assess its efficiency as a barrier against potential bloom-related toxins. Many cyano-metabolites contain functional groups such as olefins or phenols that have known reactivity with O_3_,^9,15^ which can help predicting their abatement during ozonation. However, a given moiety can have significant variability in reactivity depending on the pH and substituents.^9^ One case in point is moieties such as phenols or amines, for which the O_3_ reactivity is pH-dependent because of their acid–base speciation, controlled by the p*K*_a_ values. The p*K*_a_ can shift depending on the neighboring functional groups and is often challenging to accurately predict.^16^ In addition, little to no information is available for some functional groups such as heterocycles present in cyano-metabolites. Apparent second-order rate constants (*k*_app,O3_) of the reactions between a target compound and O_3_ can be obtained in pure water by various direct and indirect methods, including competition kinetics, in the presence of an appropriate reference compound (competitor) and a hydroxyl radical (^•^OH) scavenger (e.g., *tert*-butanol).^9,17^ However, the vast majority of cyano-metabolites are not commercially available and would have to be isolated from laboratory-grown cyanobacteria cultures, which is a major obstacle to studying the ozonation of individual cyano-metabolites.

To overcome this shortcoming, the aim of this study was to develop a novel multicompound competition kinetic approach to determine *k*_app,O3_ in mixtures of cyano-metabolites. Sixteen competitor compounds, mostly micropollutants with various functional groups, were selected to cover a wide range of partially overlapping second-order rate constants (1 to 10^8^ M^–1^ s^–1^). Regardless of the complexity of the matrix, the abatements of two given compounds (here between a cyano-metabolite and a competitor) are correlated because they are subjected to the same O_3_ exposure.^9^ Ozonation experiments in the presence of the competitors and cyano-metabolite mixtures from two strains, *Microcystis aeruginosa* PCC7806 and *Planktothrix rubescens* K-0576, were performed at pH 7 and 8 in the presence of an ^•^OH scavenger to determine *k*_app,O3_ for 31 metabolites. This was possible by measuring simultaneously the relative abatement of cyano-metabolites and competitors by a previously developed LC-HRMS/MS method.^18,19^ Several *k*_app,O3_ per cyano-metabolites could be measured using several competitors, providing a more robust determination and preventing biased values.

## Materials and Methods

### Standards and Reagents

The purity and suppliers of all chemicals and solvents are provided in Table S1 (Supporting Information SI1). Aerucyclamide A was previously isolated from *Microcystis aeruginosa* PCC7806, purified, and kept in DMSO.^20^ Stock solutions of the competitors (10 mM) were prepared in acetone or ultrapure water (Arium Pro, Sartorius, 18.7 MΩ cm). O_3_ stock solutions were prepared by sparging O_3_/O_2_ gas mixtures produced by an O_3_ generator (BMT 803 BT, BMT Messtechnik, Berlin, Germany) in ultrapure water cooled in an ice bath. The concentration of the O_3_ stock solution (0.9–1.3 mM) was measured spectrophotometrically at 260 nm in a 1 cm quartz cuvette (ε_260_ = 3200 M^–1^ cm^–1^).^9^

### Cyanobacterial Cultures and Extraction

*Microcystis aeruginosa* PCC7806 and *Planktothrix rubescens* K-0576 were obtained from the Pasteur Culture Collection (PCC) and the Norwegian Culture Collection of Algae (NORCCA), respectively. Cyanobacteria were cultivated, and their biomass extracted and purified as previously described.^21^ Details are provided in Text S1. Cyano-metabolites were stored in MeOH/H_2_O (85/15 v/v) at −20 °C for up to a month without significant degradation (Figure S1a). An aliquot of the extract was mixed with an equal volume of pure water, evaporated to dryness under vacuum (Syncore Analyst R-12, BÜCHI Labortechnik AG, 55 °C, 60 rpm, 80 min at 150 mbar, 20 min at 90 mbar, and 80 min at 20 mbar) and redissolved in ultrapure water. No loss of identified cyano-metabolites was observed during the evaporation to dryness (Figure S1b). This approach guaranteed the absence of MeOH during ozonation, which is a promotor for ^•^OH formation during ozonation.^9^

### Cyano-metabolite Analysis and Identification

Cyano-metabolites were analyzed by HPLC (Dionex Ultimate 3000 RS pump, Thermo Fischer Scientific) with a Kinetex C18 column (2.6 μm, 2.1 × 100 mm, with SecurityGuard ULTRA precolumn, Phenomenex), coupled to a high-resolution tandem mass spectrometer (HRMS/MS, Exploris, ThermoFisher Scientific).^18,19^ Details for the HPLC and HRMS/MS methods are provided in Text S2. Elution was carried out using a gradient with MeOH and ultrapure water both acidified with 0.1% formic acid. HRMS/MS used electrospray ionization (ESI) with both positive and negative ionization modes at 3.5 and 2.5 kV, respectively.

Data evaluation and peak area extraction was performed with Skyline 22.2 (MacCoss Lab). The ion chromatograms were screened with a suspect list obtained from CyanoMetDB (Version 02, 2023),^14^ searching for the [M – 2H]^2–^, [M – H]^−^, [M + H]^+^, and [M + 2H]^2+^ ions.^13,14^ Suspects were considered only if they fulfilled the following criteria: (i) exact mass < 4 ppm, (ii) isotope dot product > 0.9, and (iii) peak area ≥ 10^7^. Then, suspects were further considered if their retention times matched their expected polarities and if good MS^2^ spectra were obtained. The final list of cyano-metabolites is provided in Table S4. Since no spectral libraries exist for most cyano-metabolites, in-silico fragmentation predictions were used to facilitate manual compound annotation of MS^2^ spectra (MetFrag Web and Sirius 4.4).^22,23^ Suspected structures were manually evaluated, prioritizing fragmentation around the peptide bonds (details are given in Text S3). Only compounds identified with a confidence level of at least 3 were used (see Table S4).^24^ The MS^2^ annotations and, when possible, head-to-tail MS^2^ comparisons with standards or bioreagents are available in a separate data spreadsheet (SI2).

### Ozonation Experiments

Kinetic studies were performed at room temperature (22 ± 1 °C), using cyano-metabolite mixtures extracted from 0.8 to 1.2 g_biomass_ L^–1^ of *Planktothrix* or *Microcystis*, in the presence of competitors (at 0.4 μM each), phosphate buffer (4 mM, pH 7 or 8), and *tert*-butanol (80 mM). The dissolved organic carbon concentrations of the samples (excluding *tert*-butanol) were about 12–16 mgC L^–1^. The cyano-metabolite and competitor mixture was split in 1 mL aliquots to which 1 mL of prediluted O_3_ was added under vigorous stirring. O_3_ was prediluted in pure water from the O_3_ stock to prevent the spiking of samples with small volumes of highly concentrated O_3_, leading to strong concentration gradients and potentially undesired side reactions. The O_3_ doses were in the range of 0.1–90 μM after dilution (0.01–7.9 mgO_3_/g_biomass_). Experiments were performed in triplicate on three separate days.

### Determination of Second-Order Rate Constants

Competition kinetics were applied to determine the *k*_app,O3_ of cyano-metabolites (*k*_app,O3,cyanomet_):^9^
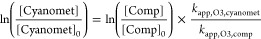
1

Instead of concentrations, peak areas can directly be used (as long as the response is linear) because only the relative abatement needs to be known. *k*_app,O3,cyanomet_ can be derived from the linear regression slope of the ln of the cyano-metabolite relative residual peak area as a function of the ln of the competitor relative residual peak areas. *k*_app,O3,comp_ values are provided in Table 1 for pH 7. For competitors undergoing acid–base speciation, *k*_app,O3,comp_ is pH-dependent and was calculated at a given pH by eq 2:
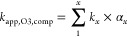
2with *x* being the number of species, *k*_*x*_ the second-order rate constant for the reaction of a given species with O_3_ (Table S3), and α_*x*_ the molar fraction of the given species at a given pH.

**Table 1:** List of 16 Selected Competitors with Their *k*_app,O3_ Values at pH 7^a^

Competitor (abbreviation)	*k*_app,O3_ at pH 7 (M^–1^ s^–1^)	Competitor (abbreviation)	*k*_app,O3_ at pH 7 (M^–1^ s^–1^)
Acetylsulfamethoxazole (ASMX)	(2.5 ± 0.1) × 10^2^ (ref ^17^)	Penicillin G (PG)	(4.8 ± 0.1) × 10^3^ (ref ^17^)
Alachlor (ALA)	3.8 ± 0.4 (ref ^25^)	Picloram (PCL)	(1.4 ± 0.2) × 10^2^ (ref ^25^)
Bezafibrate (BZF)	(5.9 ± 0.5) × 10^2^ (ref ^26^)	Roxithromycin (ROX)	(6.3 ± 1.4) × 10^4^ (ref ^17^)
Carbamazepine (CBZ)	(6.1 ± 0.1) × 10^5^ (ref ^27^)	Sulfamethoxazole (SMX)	(1.1 ± 0.2) × 10^6^^e^ (ref ^17^)
Carbofuran (CBF)	6.2 × 10^2^ (ref ^25^)	Tramadol (TRA)	(4.0 ± 0.9) × 10^3^ (ref ^28^)
	2.1 × 10^2^^c^		
Ciprofloxacin (CIP)	(1.9 ± 0.7) × 10^4^ (ref ^17^)	Triclosan (TRI)	(3.8 ± 0.8) × 10^7^ (ref ^29^)
Diazepam (DZP)	(7.5 ± 0.15) × 10^–1^ (ref (26))	Trimethoprim (TMP)	(5.4 ± 1.1) × 10^5^^e^ (ref ^17^)
Dibromomethylparaben^b^ (DMP)	8.4 × 10^7^ (ref ^30^)	Tylosin (TYL)	(1.0 ± 0.2) × 10^5^^e^ (ref ^17^)
	(4.3 ± 0.3) × 10^6^^d^		

a The structures and species-specific second-order rate constants for the reactions with O_3_ are shown in Table S3.

b IUPAC name: methyl 3,5-dibromo-4-hydroxybenzoate.

c Second-order rate constant corrected for stoichiometry. See explanations in section Validation of Competitors.

d Second-order rate constants redetermined using cinnamic acid and phenol as competitors. See details in section Validation of Competitors and Text S4.

e Second-order rate constants recalculated based on the re-evaluated second-order rate constant for cinnamic acid by Kim et al.^31^

Examples of correlation plots between relative abatements of cyano-metabolites and competitors are shown in Figure S4. Only data points with a relative abatement of cyano-metabolites and competitors between 10% and 90% were considered. There were two main reasons for this approach: (i) a wide range of O_3_ doses was applied and each compound only reacted at a specific range of O_3_ doses. For compounds with intermediate or low reactivity, many data points at negligible abatement would overly impact the linear regression, which was avoided by setting a minimum of 10% abatement. (ii) The 90% maximum abatement limit was set to avoid loss of linearity, prevent high leverage data points and eventual interference due to carryover between sample injections on the HPLC-HRMS/MS.

*k*_app,O3_ for a given cyano-metabolite/competitor pair was only calculated if (i) there were 10 or more data points, (ii) the coefficient of determination (*R*^2^) was higher than 0.9, (iii) the intercept was negligible (intercept < 10 × slope), and (iv) the slope was between 0.1 and 10 (i.e., if the *k*_app,O3_ for the cyano-metabolite and the *k*_app,O3_ for the competitor were within 1 order of magnitude). The statistical parameters of each successful linear regression are provided in Tables S5–S10. For a given cyano-metabolite/competitor pair, the standard error (SE) of the cyano-metabolite *k*_app_ (SE_cyanomet/comp_) was the result of the propagation of the standard error on the slope (SE_slope_) and on the *k*_app,O3_ of the competitor (SE_comp_):
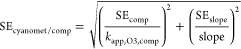
3

The uncertainty in *k*_app,O3,comp_ given in the literature was used for SE_comp_. If no error was provided in the literature for the *k*_app,O3_ of the competitor, a 20% relative error was set (most *k*_app,O3,comp_ values have relative errors of 20% or less). For competitors with acid–base speciation, the error of *k*_app,O3_ in the literature was replaced by the error induced from ±0.05 pH variation, if the latter was greater. This was the case for roxithromycin, tramadol, and triclosan at pH 7.

For a given cyano-metabolite, several *k*_app,O3_ could be obtained from several competitors (e.g., anabaenopeptin A correlated with trimethoprim, carbamazepine, tylosin, sulfamethoxazole and dibromomethylparaben, each giving a *k*_app,O3_). In addition, the same cyano-metabolite could be monitored at different ionizations (given in Table S4) in HRMS/MS (e.g., the [M + H]^+^ and [M – H]^−^ ions of anabaenopeptin A were simultaneously monitored). Different ionizations did not significantly impact the *k*_app,O3_ (<20% difference, *k*_app,O3_ for individual ionizations are not shown). All the *k*_app,O3_ determined for a given cyano-metabolite were averaged and the standard error was either the standard deviation of all the *k*_app,O3_ or the highest competitor-specific *k*_app,O3_ standard error, whichever was the highest.

## Results and Discussion

### Identification of Cyano-metabolites in Microcystis and Planktothrix Extracts

**Figure 1:**
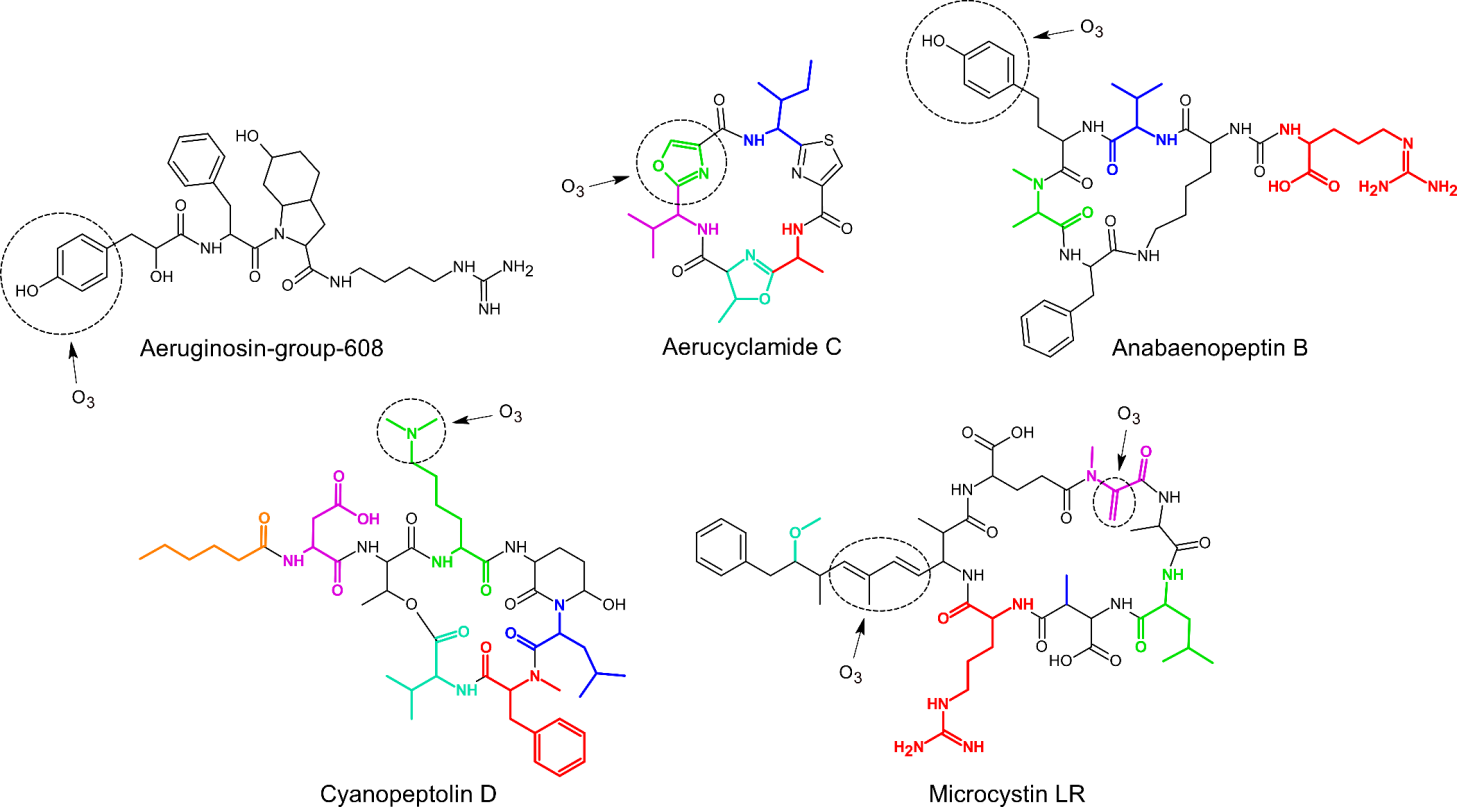
Representative cyano-metabolites detected in *Microcystis aeruginosa* and *Planktothrix rubescens* cultures. Highlighted moieties represent the parts of the molecules that can vary in the other cyano-metabolites of the same class identified in this study. Circles indicate the main attack sites of the O_3_. The full lists of cyano-metabolites and their structures are provided in Table S4, Tables S11–S14, and Figure S5.

A total of 31 cyano-metabolites with sufficient signal intensity for kinetic studies were identified from the selected strains: 1 aeruginosin, 5 anabaenopeptins, 7 cyanopeptolins, 6 cyclamides, 10 microcystins, and 2 unclassified cyano-metabolites.^32^Figure 1 shows the structures of representative cyano-metabolites for each class. Highlighted moieties represent the parts of the molecule that can change for other identified variants of the same class. The full cyano-metabolite list with their structures is provided in the SI (Table S4, Tables S11–S14, and Figure S5).

Aeruginosin-group-608 is the only noncyclic cyano-metabolite identified in this study. It refers to one of the three known stereoisomers of the shown structure (Figure 1). Aeruginosins are tetrapeptides notably characterized by a lactic acid derivative that contains a phenol, which is expected to be a major reactive site for O_3_.^33^

Cyclamides, represented by aerucyclamide C in Figure 1, are cyclic hexapeptides characterized by heterocyclic groups such as thiazole, oxazole, thiazoline, and oxazoline that have unknown reactivities toward O_3_. In our study, oxazole was determined to be the main reactive site in aerucyclamide C (see explanation in “Determination of Second-Order Rate Constants for the Reactions of Cyano-metabolites with Ozone”). One cyclamide, aerucyclamide D, contains a methionine, which is expected to be a major reactive site (Table S13).^34^

Anabaenopeptins are characterized by a cyclic pentapeptide containing a lysine. The lysine’s α-amine branches out to form a urea bond with the N-terminal of a sixth amino acid outside of the cycle (an arginine in the case of anabaenopeptin B, Figure 1). All the anabaenopeptins identified in this study contain a homotyrosine and eventually a tyrosine, which are expected to be the major reactive sites for O_3_.^34^

Cyanopeptolins are characterized by a cyclic hexadepsipeptide containing a 3-amino-6-methoxy-2-piperidone and a threonine that forms an ester bond (Figure 1). In addition, the N-terminus of the threonine branches out to two supplementary amino acids. The main O_3_ attack site in cyanopeptolin D is expected to be the tertiary amine on the side chain of the lysine derivative. In other cyanopeptolins, the lysine derivative is replaced by other amino acids such as a normal lysine or a tyrosine, which are also expected to be major reactive sites (Table S12).^34,37^

Microcystins (abbreviated as MC) are cyclic heptapeptides with a characteristic Adda moiety (3-amino-9-methoxy-2,6,8-trimethyl-10-phenyl-deca-4,6-dienoic acid) (Figure 1). Adda contains conjugated olefins that are a major reactive site for O_3_.^12,31^ One microcystin, [Mdha-GSH7]MC-LR, contains an additional thioether that is expected to be more reactive than the conjugated olefins (Figure S5).^34^

Two additional cyclic cyano-metabolites, identified in *Planktothrix*, are planktocyclin and piricyclamide ILGEGEGWNYNP+prenyl (Figure S5). They contain a methionine and tryptophan, respectively, which are expected to be the main reactive sites.^34^

### Validation of Competitors

**Figure 2:**
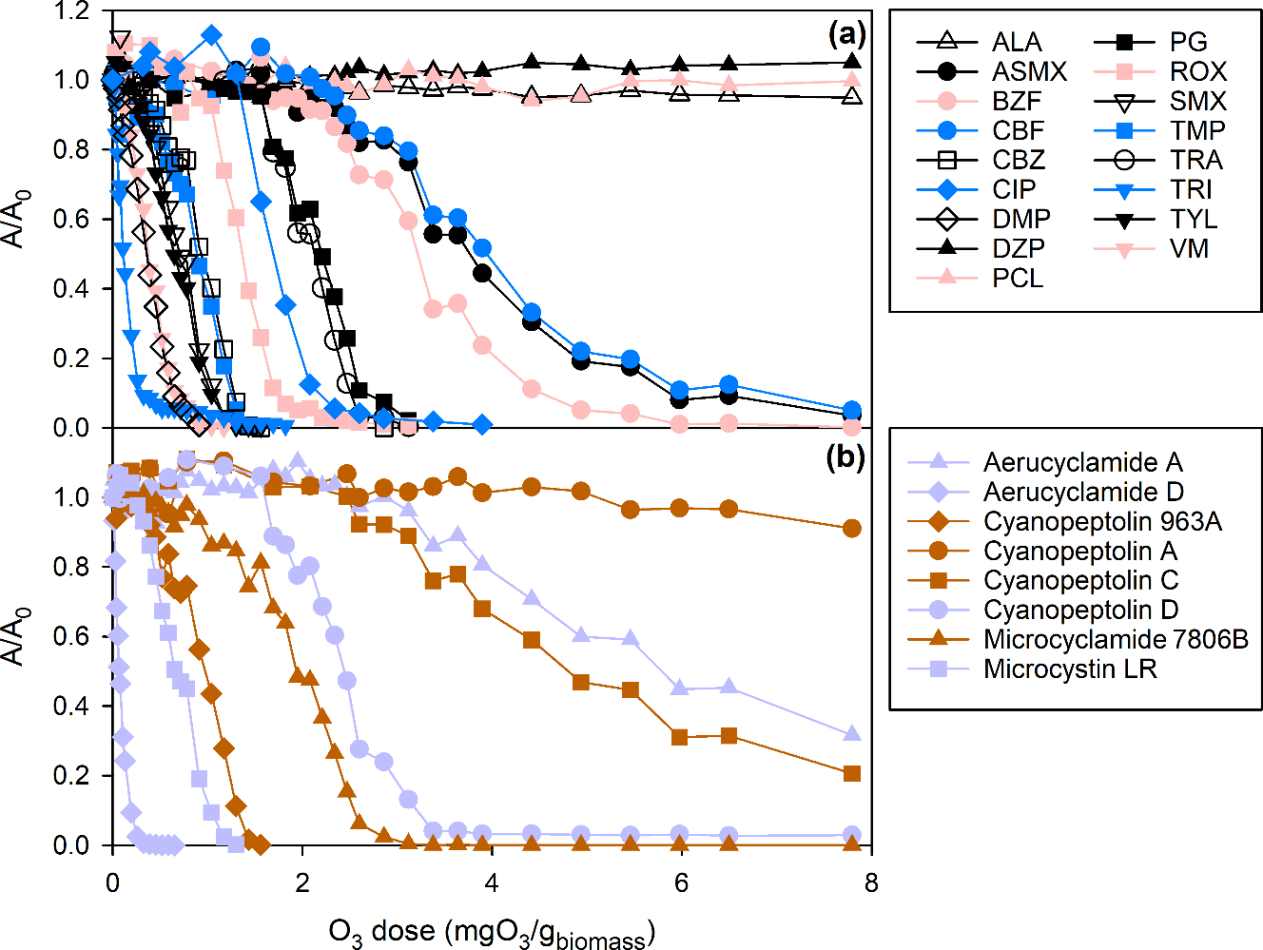
Simultaneous abatement of (a) the selected competitors and (b) representative cyano-metabolites from 0.6 g_biomass_ L^–1^ of *Microcystis* as a function of the specific O_3_ dose at pH 7 (2 mM phosphate) and 22 °C and in the presence of *tert*-butanol (40 mM). For the abbreviations of the competitors, see Table 1 (except VM which stands for vancomycin).

First, the abatement of competitors and their previously published *k*_app,O3_ values were evaluated in the presence of cyano-metabolite mixtures extracted from the two selected cyanobacteria strains. The abatement of the selected competitors as a function of the O_3_ dose in the presence of the cyano-metabolite mixture from *Microcystis* at pH 7 is shown in Figure 2a. The abatement of the competitors was generally consistent with their reactivity: a higher *k*_app,O3_ led to a higher abatement for a specific ozone dose. However, a few exceptions were observed: dibromomethylparaben (DMP, *k*_app,O3_ = 8.4 × 10^7^ M^–1^ s^–1^) should be more reactive than triclosan (TRI) and vancomycin (VM, not shown in Table 1) (3.8 × 10^7^ and 1.2 × 10^6^ M^–1^ s^–1^, respectively).^17,29,30^ However, Figure 2a shows that DMP (open diamonds) required higher or similar O_3_ doses than TRI (reverse blue triangles) or VM (reverse red triangles) for an equivalent abatement. Likewise, carbofuran (CBF, blue circles) required higher O_3_ doses than benzafibrate (BZF, red circles) to reach an equivalent abatement, while their reported *k*_app,O3_ values are comparable (6.2 × 10^2^ and 5.9 × 10^2^ M^–1^ s^–1^, respectively).^25,26^

**Figure 3:**
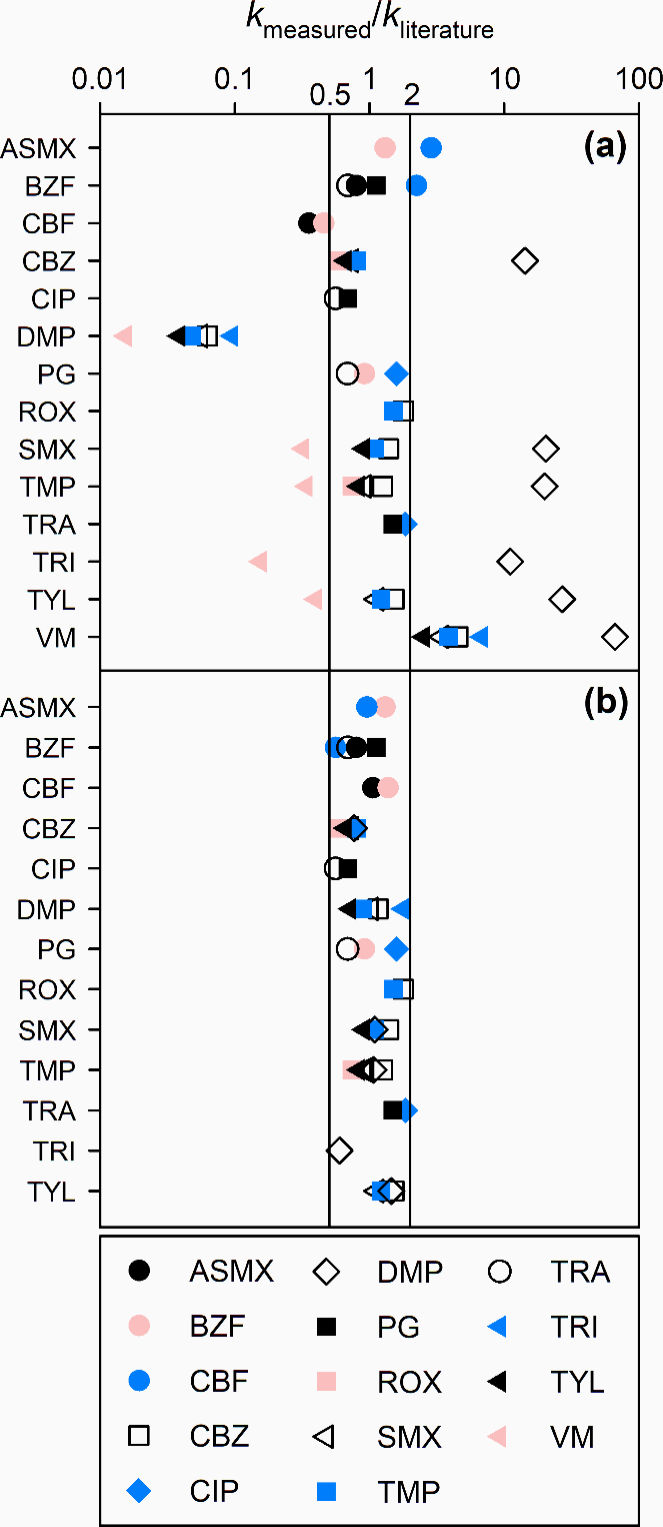
Competitor evaluation during the ozonation of a *Microcystis* extract (0.6 g_biomass_ L^–1^) at pH 7 (2 mM phosphate) and 22 °C and in the presence of *tert*-butanol (40 mM). The evaluation was done by calculating the ratios between the *k*_app,O3_ determined by pairs of competitors (*k*_measured_) and the *k*_app,O3_ from the literature (*k*_literature_, see Table 1). Panels (a) and (b) show the *k*_measured_/*k*_literature_ ratios using unmodified and adjusted *k*_literature_, respectively (only *k*_literature_ of CBF and DMP were adjusted; see explanation in the text). The vertical lines correspond to the limits for the acceptable *k*_measured_/*k*_literature_ range, set between 0.5 and 2. For the abbreviations of the competitors see Table 1 (except VM which stands for vancomycin).

To evaluate whether the observed discrepancies were significant, competitors were systematically compared to each other, using eq 1. The abatement of a given competitor was correlated with the other competitors with similar reactivities, i.e., leading to regression slopes between 0.1 and 10, from which apparent second-order rate constants were calculated (*k*_measured_). Figure 3a shows for each competitor the ratios between *k*_measured_ in the *Microcystis* extract at pH 7 and the previously published apparent second-order rate constant (*k*_literature_, shown in Table 1). For example, for penicillin G (PG), three symbols are shown, which represent three *k*_measured_/*k*_literature_ ratios using tramadol (TRA, open circle), BZF (filled red circle), and ciprofloxacin (CIP, filled blue diamond) as competitors. The *k*_measured_/*k*_literature_ ratios for PG were between 0.7 and 1.4, which is in the range of variations for second-order rate constants from different studies. Overall, the majority of *k*_measured_/*k*_literature_ ratios were between 0.5 and 2, which is an acceptable variation range for competition kinetics (Figure 3a). However, using carbofuran (CBF), VM, or DMP as competitors consistently led to ratios beyond a 2-fold difference, suggesting that their *k*_literature_ could be inaccurate, as discussed in more detail in the following. For diazepam (DZP), alachlor (ALA), and picloram (PCL) no *k*_measured_/*k*_literature_ values were determined as they were not significantly degraded at the applied O_3_ doses (Figure 2a).

#### Carbofuran (CBF)

The *k*_measured_ values of CBF with acetylsulfamethoxazole (ASMX) or BZF as competitors were 2 to 3-fold lower than its *k*_literature_ (black and red filled circles on the CBF line, Figure 3a). Conversely, the *k*_measured_ values of ASMX and BZF with CBF as competitor were 2 to 3-fold higher than their *k*_literature_ (blue filled circles, Figure 3a). This discrepancy was attributed to the determination method of *k*_O3,CBF_. *k*_O3,CBF_ (6.2 × 10^2^ M^–1^ s^–1^) was previously measured by monitoring the O_3_ decrease in excess of CBF.^25^ However, the O_3_:CBF reaction stoichiometry was determined in our study to be 3:1 (see Figure S6), implying that *k*_O3,CBF_ related to CBF abatement has to be divided by a stoichiometric factor of 3, resulting in a corrected *k*_O3,CBF_ of 2.1 × 10^2^ M^–1^ s^–1^. Using the corrected *k*_O3,CBF_, the *k*_measured_/*k*_literature_ ratios involving CBF improved from 0.3–2.9 to 0.8–1.4 (Figure 3b).

#### Vancomycin (VM)

The *k*_measured_ values of VM determined with trimethoprim (TMP), tylosin (TYL), sulfamethoxazole (SMX), and TRI as competitors were 3 to 7-fold higher than its *k*_literature_ (symbols on the VM line, Figure 3a). Conversely, the *k*_measured_ values of TMP, TYL, SMX, and TRI determined with VM as competitor were 2 to 8-fold lower than their *k*_literature_ (red filled triangles, Figure 3a). VM contains a resorcinol, a phenol, and amine groups that serve as secondary reaction sites. Due to the complexity of VM (six p*K*_a_ values and three activated aromatic rings) no species-specific second-order rate constants could previously be determined and only *k*_app,O3_ values were available, which may have caused such a discrepancy.^17^ Because of this complexity, *k*_O3,VM_ was not reassessed, omitted from Figure 3b, and not considered further in this study.

#### Dibromomethylparaben (DMP)

The *k*_measured_ values of DMP determined with TMP, CBZ, TYL SMX, and TRI as competitors were more than 10-fold lower than the reported literature value (symbols on the DMP line, Figure 3a). Conversely, the *k*_measured_ values of TMP, CBZ, TYL, SMX, and TRI determined with DMP as competitor were 10 to 25-fold higher than the reported values (open diamonds, Figure 3a). *k*_O3,DMP_ of the deprotonated DMP (8.4 × 10^7^ M^–1^ s^–1^) has previously been determined by competition kinetics with indigotrisulfonate as competitor at pH ≥ 7.^30^ The *k*_O3_ of indigotrisulfonate has been determined with 1,3,5-trimethoxybenzene as competitor and the *k*_O3_ of the latter with buten-3-ol as competitor.^38^ This cascade for determinations of second-order rate constants might have led to accumulated errors. In the present study, *k*_O3,DMP_ was redetermined using cinnamic acid and phenol as competitors, for which directly measured *k*_O3_ are available.^31,33^ A corrected *k*_O3,DMP_ = (4.3 ± 0.3) × 10^6^ M^–1^ s^–1^ was measured for the deprotonated DMP, 19-fold lower than the previous literature value (details are provided in Text S4). The *k*_O3_ for protonated DMP was not re-evaluated as it is expected to be negligible in the studied pH range (p*K*_a_ = 4.7). Using the corrected *k*_O3,DMP_, the *k*_measured_/*k*_literature_ ratios involving DMP improved from 0.04–27 to 0.7–1.8 (Figure 3b).

#### Validation in Planktothrix Mixture and at pH 8

After correcting the apparent *k*_O3,CBF_ and *k*_O3,DMP_ values and excluding VM, all the *k*_measured_ were within a factor of 2 of the *k*_literature_ at pH 7 and therefore the selected competitors were suitable for this study. A similar exercise was done in the presence of the cyano-metabolite mixture from *Planktothrix* at pH 7 and 8, but only for competitors with a *k*_app,O3_ > 10^4^ M^–1^ s^–1^ (see Figure S7). Again, all the *k*_measured_ values were within a factor of 2 of the *k*_literature_ values, excluding VM, further validating the competitor *k*_app,O3_, especially for the pH-dependent competitors ROX, TMP, and TYL. For these three competitors, due to p*K*_a_ values between 7.1 and 9.2 and *k*_O3,neutral_ > *k*_O3,protonated_ (see Table S3), their *k*_app,O3_ values increase 1.7 to 9.5-fold when increasing the pH from 7 to 8. This pH effect can be a supplementary source of error when determining *k*_app,O3_ due to added uncertainties on pH control, p*K*_a_, and model fitting. The *k*_app,O3_ of TRI is also pH-dependent, but it was too reactive to be correlated with any of the other competitors at pH 8.

### Determination of Second-Order Rate Constants for the Reactions of Cyano-metabolites with Ozone

The abatement of representative cyano-metabolites as a function of the specific O_3_ dose is shown in Figure 2b. The abatement of each cyano-metabolite was correlated to the abatement of the competitors with similar reactivity. Examples of abatement correlation plots used for the determination of *k*_app,O3_ of cyano-metabolites are given in Figure S4. A summary of the averaged *k*_app,O3_ of cyano-metabolites at pH 7 determined with multiple competitors is provided in Table 2, classified by their main reactive moieties. Competitor-specific *k*_app,O3_ values are provided for all cyano-metabolites in Table S16. Overall, of the 31 metabolites, three did not react with O_3_ at a measurable rate (*k*_app,O3_ < 10^2^ M^–1^ s^–1^), six metabolites reacted slowly/moderately with *k*_app,O3_ ≤ 5.0 × 10^3^ M^–1^ s^–1^, 19 metabolites reacted readily with *k*_app,O3_ ranging between 3.6 × 10^5^ and 1.9 × 10^6^ M^–1^ s^–1^, and three metabolites reacted rapidly with *k*_app,O3_ ranging between 3.4 and 7.3 × 10^7^ M^–1^ s^–1^. The structure–reactivity dependency for the reaction of these metabolites with O_3_ is discussed in the following.

**Table 2:** Apparent Second-Order Rate Constants (*k*_app,O3_) at pH 7 for the Reactions of O_3_ with Cyano-metabolites from *Microcystis aeruginosa* (PCC7806) and *Planktothrix rubescens* (K-0576)^a^

Cyano-metabolites	Cyanobacterial strain	*k*_app,O3_ at pH 7 (M^–1^ s^–1^)^d^	Competitors
*Tryptophan*			
Piricyclamide ILGEGEGWNYNP + prenyl	*Planktothrix*	(7.3 ± 1.7) × 10^7^	TRI
*Thioethers*			
[Mdha-GSH7]MC-LR	*Microcystis*	(1.9 ± 0.4) × 10^6^	TMP, TYL, SMX, DMP
Aerucyclamide D	*Microcystis*	(5.2 ± 1.5) × 10^7^	DMP, TRI
Planktocyclin	*Planktothrix*	(3.4 ± 0.9) × 10^7^	DMP, TRI
*Olefins*			
[d-Asp3,(E)-Dhb7]MC-RR	*Planktothrix*	(1.7 ± 0.3) × 10^6^	TMP, CBZ, TYL, SMX, DMP
[d-Asp3,Dha7]MC-RR	*Planktothrix*	(1.3 ± 0.3) × 10^6^	TMP, CBZ, TYL, SMX, DMP
[d-Asp3,DMAdda5]MC-RR	*Planktothrix*	(1.7 ± 0.3) × 10^6^	TMP, CBZ, TYL, SMX, DMP
[d-Asp3]MC-LA	*Planktothrix*	(9.7 ± 2.1) × 10^5^	TMP, CBZ, TYL, SMX
[d-Asp3]MC-LR	*Microcystis*, *Planktothrix*	(1.1 ± 0.2) × 10^6^, (1.1 ± 0.2) × 10^6^	TMP, CBZ, TYL, SMX, DMP
MC-HilR	*Microcystis*	(1.1 ± 0.2) × 10^6^	TMP, CBZ, TYL, SMX, DMP
MC-LAba	*Planktothrix*	(1.1 ± 0.2) × 10^6^	TMP, CBZ, TYL, SMX
MC-LR	*Microcystis*, *Planktothrix*	(1.1 ± 0.2) × 10^6^, (1.1 ± 0.2) × 10^6^	TMP, CBZ, TYL, SMX, DMP
Microcystin-group-967	*Microcystis*	(1.1 ± 0.2) × 10^6^	TMP, CBZ, TYL, SMX
*Phenols*			
Aeruginosin-group-608	*Planktothrix*	(1.6 ± 0.3) × 10^6^	TMP, CBZ, TYL, SMX, DMP
Anabaenopeptin A	*Planktothrix*	(1.2 ± 0.2) × 10^6^	TMP, CBZ, TYL, SMX, DMP
Anabaenopeptin B	*Planktothrix*	(9.7 ± 1.9) × 10^5^	TMP, CBZ, TYL, SMX
Anabaenopeptin F	*Planktothrix*	(1.0 ± 0.2) × 10^6^	TMP, CBZ, TYL, SMX
Anabaenopeptin SA13	*Planktothrix*	(1.2 ± 0.2) × 10^6^	TMP, CBZ, TYL, SMX
Cyanopeptolin 1020	*Planktothrix*	(5.7 ± 1.2) × 10^5^	TMP, CBZ, TYL, SMX
Cyanopeptolin 963A^b^	*Microcystis*	(3.6 ± 0.8) × 10^5^	TMP, CBZ, TYL, SMX
Oscillamide Y	*Planktothrix*	(1.2 ± 0.3) × 10^6^	TMP, CBZ, TYL, SMX
Oscillapeptin J	*Planktothrix*	(1.6 ± 0.4) × 10^6^	TMP, CBZ, TYL, SMX, DMP
*Amines*			
Cyanopeptolin B^b^^,^^c^	*Microcystis*	<10^2^	
Cyanopeptolin C^b^^,^^c^	*Microcystis*	(1.0 ± 0.3) × 10^2^	ASMX, BZF, CBF, TRA, PG
Cyanopeptolin D^b^	*Microcystis*	(2.0 ± 0.5) × 10^3^	ASMX, BZF, CBF, TRA, PG
*Heterocycles*			
Aerucyclamide A	*Microcystis*	(8.8 ± 1.6) × 10^1^	ASMX, BZF, CBF
Aerucyclamide B	*Microcystis*	<10^2^	
Aerucyclamide C	*Microcystis*	(4.2 ± 1.1) × 10^3^	TRA, PG
Microcyclamide 7806A	*Microcystis*	(4.7 ± 1.2) × 10^3^	BZF, TRA, PG
Microcyclamide 7806B	*Microcystis*	(5.0 ± 1.1) × 10^3^	BZF, TRA, PG
*Benzene*			
Cyanopeptolin A^b^	*Microcystis*	<10^2^	

a The *k*_app,O3_ values were determined by competition kinetics with the indicated competitors. For abbreviations of competitors, see Table 1.

b Cyano-metabolites for which an isomer with the same MS^2^ fragmentation was found within 1 min of retention time. *k*_app,O3_ for the two isomers were within ±20%.

c The *k*_app,O3_ of these cyano-metabolites is to be taken with caution. Cyanopeptolin C is a potential product, yet minor, of cyanopeptolin D ozonation and cyanopeptolin B a potential product, yet minor, of cyanopeptolin C ozonation (see explanation in text and in Text S5).

d Whenever a cyano-metabolite was present in the two strains, two *k*_app,O3_ are reported.

#### Tryptophan and Thioether

Tryptophan- and thioether-containing cyano-metabolites were the most reactive toward O_3_ with *k*_app,O3_ at pH 7 in the range 3.4–7.3 × 10^7^ M^–1^ s^–1^, excluding [Mdha-GSH7]MC-LR (Table 2). These *k*_app,O3_ were an order of magnitude higher than reported *k*_app,O3_ values for free tryptophan (7.0 × 10^6^ M^–1^ s^–1^) and methionine (4.0 × 10^6^ M^–1^ s^–1^).^34^ In the previous study, *k*_app,O3_ values for free tryptophan and methionine were measured with histidine or 3-hexenoic acid as competitors for which *k*_app,O3_ are significantly lower (1.9 × 10^5^ and 2.4 × 10^5^ M^–1^ s^–1^, respectively, at pH 7).^34^ This large difference in *k*_app,O3_ is not ideal for competition kinetics and may lead to high errors. Therefore, the *k*_O3_ values of these two amino acids need to be redetermined in future studies. The *k*_app,O3_ obtained for [Mdha-GSH7]MC-LR (1.9 × 10^6^ M^–1^ s^–1^) was much lower than those obtained for aerucyclamide D and planktocyclin (Table 2). In [Mdha-GSH7]MC-LR, the thioether is attached to the microcystin ring on one side and to the rest of the glutathione on the other side (Figure S5). This position may cause a decrease in O_3_ reactivity of the thioether in [Mdha-GSH7]MC-LR. Large variations in the reactivity of thioethers have previously been observed for pharmaceuticals.^17^ The measured *k*_app,O3_ for [Mdha-GSH7]MC-LR was similar to that of other microcystins (Table 2). It is therefore uncertain whether the main attack site for O_3_ is the thioether or the olefins. Further investigation of the effect of complex substituents on the thioether reactivity is required.

#### Olefins and Phenols

The *k*_app,O3_ of olefin-containing cyano-metabolites ranged between 1.0 and 1.7 × 10^6^ M^–1^ s^–1^, somewhat higher than the *k*_O3_ value of the protonated form of sorbic acid (3.7 ± 0.3 × 10^5^ M^–1^ s^–1^, adjusted with the re-evaluated *k*_cinnamic acid_).^12,31^ In addition, the same *k*_app,O3_ for MC-LR was calculated in *Microcystis* and *Planktothrix* ((1.1 ± 0.2) × 10^6^ M^–1^ s^–1^), consistent with a previous study ((8.5 ± 0.2) × 10^5^ M^–1^ s^–1^).^31^ It is worth noting that MC-LR is one of the main cyano-metabolites in *Microcystis* while it is a minor cyano-metabolite in *Planktothrix* (about 50 times less concentrated), further demonstrating that the matrix does not interfere with the determination of *k*_app,O3_. Phenol-containing cyano-metabolites had *k*_app,O3_ ranging between 0.4 and 1.6 × 10^6^ M^–1^ s^–1^, consistent with the reactivity of phenol (1.8 ± 0.5 × 10^6^ M^–1^ s^–1^).^33^ In addition, cyano-metabolites containing two phenols (e.g., anabaenopeptins A and SA13) were not significantly more reactive than those with one phenol (e.g., anabaenopeptins B and F).

#### Amines

Three cyano-metabolites contain amines: cyanopeptolin B, cyanopeptolin C, and cyanopeptolin D. These three cyanopeptolins have the same structure, apart from the amine-containing moieties, which are expected to be the main reactive sites (Table S12). Cyanopeptolin B contains a primary amine on the side chain of a lysine. Cyanopeptolins C and D contain lysine derivatives, in which the side chain amine is substituted by one and two methyls, respectively. The reactivity order of these three cyanopeptolins at pH 7 was cyanopeptolin D (tertiary amine) > cyanopeptolin C (secondary amine) > cyanopeptolin B (primary amine), consistent with the ozonation literature on amines (Table 2).^37^ However, the *k*_app,O3_ of the tertiary amine-containing cyanopeptolin D ((2.0 ± 0.5) × 10^3^ M^–1^ s^–1^) was an order of magnitude higher than the *k*_app,O3_ of triethylamine reported in the literature ((2.2 ± 0.1) × 10^2^ M^–1^ s^–1^).^37^ This difference may be, in part, due to a lower p*K*_a_ for cyanopeptolin D compared with that of triethylamine. The predicted p*K*_a_ of cyanopeptolin D was 0.5 pH-unit lower than the predicted p*K*_a_ of triethylamine (p*K*_a_ predicted by ChemAxon software, shown in Table S17). As the neutral amine is the reactive form with O_3_, a lower p*K*_a_ leads to a higher reactivity at pH 7. Another difference between cyanopeptolins and small model compounds was the extent of the decrease in reactivity between the tertiary and secondary amine. The *k*_app,O3_ of cyanopeptolin D (tertiary amine) was 20-fold higher than the *k*_app,O3_ of cyanopeptolin C (secondary amine) (Table 2). By comparison, the literature value of the *k*_app,O3_ of triethylamine (tertiary amine) is only 1.7 times higher than the *k*_app,O3_ of diethylamine (secondary amine).^37^ Again, p*K*_a_ may play a role. The predicted p*K*_a_ for cyanopeptolin C (secondary amine) was 1.2 pH-units higher than that for cyanopeptolin D (tertiary amine), while the predicted p*K*_a_ for diethylamine was only 0.4 pH-units higher than that for triethylamine (Table S17). An increased p*K*_a_ for secondary amines compared to tertiary amines can explain, in part, their lower reactivity at pH 7. However, the *k*_app,O3_ values of cyanopeptolin C and B need to be taken with caution. A possible yet minor product of tertiary amine ozonation is the corresponding secondary amine (5% from trimethylamine).^37^ Similarly, the ozonation of a secondary amine can lead to minor yields of the corresponding primary amine (8% from diethylamine).^37^ Cyanopeptolin C can therefore be formed from cyanopeptolin D ozonation, and cyanopeptolin B can be formed from cyanopeptolin C ozonation. Although no increase was observed for cyanopeptolin C and B (Figure S8), it could have been masked by their degradation during ozonation, leading to underestimated *k*_app,O3_ and to the large *k*_app,O3_ difference between cyanopeptolins D and C. However, it was estimated that no more than 10% of the initial cyanopeptolin C should be formed from cyanopeptolin D, suggesting that its *k*_app,O3_ was not significantly underestimated (see details in Text S5).

#### Heterocycles

Four cyano-metabolites contained heterocycles that likely served as primary O_3_ reaction sites. At pH 7, *k*_app,O3_ values for aerucyclamide A and B were much lower (<10^2^ M^–1^ s^–1^) compared to aerucyclamide C and microcyclamides 7806A and 7806B (4.2–5.0 × 10^3^ M^–1^ s^–1^) (Table 2). Comparing the structures of these four cyano-metabolites can help identify the reactive moieties. Oxazole was only present in the more reactive aerucyclamide C and microcyclamide 7806A and 7806B while thiazole, oxazoline, and thiazoline were present in at least one of the less reactive aerucyclamide A and B (Table S13). Altogether, this suggests that the moieties responsible for the reactivity of aerucyclamide C and microcyclamide 7806A and 7806B are oxazole. The *k*_app,O3_ of the other heterocycles are likely <10^2^ M^–1^ s^–1^ and need to be further studied.

#### pH Effect on Reaction Kinetics

The *k*_app,O3_ of cyano-metabolites present in the *Planktothrix* extract were also measured at pH 8 (Table S18). For the *k*_app,O3_ of olefin-containing cyano-metabolites, a <20% difference was observed between pH 7 and 8, consistent with their pH-independent reactivities (Tables 2 and S18). Conversely, the *k*_app,O3_ for phenol-containing cyano-metabolites increased by a factor ranging between 7 ± 2 and 9 ± 2 when increasing the pH from 7 to 8 (using *k*_app,O3_ determined with TYL, which is the only competitor that correlated at both pH values), due to the shift from protonated to deprotonated phenol moieties (Tables S16 and S18). This increase is close to the 10-fold increase of *k*_app,O3_ expected for phenolic compounds when increasing the pH from 7 to 8.^33^

For tryptophan- and thioether-containing cyano-metabolites, large discrepancies between the two competitors used at pH 8, DMP and TRI, were observed. The *k*_app,O3_ values determined with TRI were 6-fold higher than those determined with DMP (Table S18). When validating competitors, TRI was too reactive to be correlated to any other competitor at pH 8 while DMP was validated by four other competitors (see the section Validation of Competitors and Figure S7b). Hence, the *k*_app,O3_ determined with TRI at pH 8 may be overestimated. The *k*_app,O3_ of the thioether-containing planktocyclin was comparable at pH 7 and 8 when measured with DMP ((2.8 ± 0.4) × 10^7^ and (1.8 ± 0.2) × 10^7^ M^–1^ s^–1^, respectively), consistent with the expected pH-independence of thioether reactivity. For the tryptophan-containing piricyclamide ILGEGEGWNYNP+prenyl, only TRI could be correlated at pH 7 and 8; therefore the pH effect on its *k*_app,O3_ cannot be discussed.

### Practical Implications

This study demonstrates the applicability of the multicompound approach for the screening of *k*_app,O3_ for many cyano-metabolites and with a minimal set of experiments. As toxins and bioactive metabolites from cyanobacteria are usually not commercially available and can only be extracted from the producing bacteria as mixtures, multicompound competition kinetics are the most efficient way to gain knowledge on the efficiency of their abatement during ozonation. Multicompound competition kinetics are a rapid screening tool to determine the kinetics of O_3_ for a wide range of compounds at once. It is important to note that possible interferences associated with the complexity of the matrix and the measurement method exist: (1) the formation of a target compound from the oxidation of another (un)known compound, (2) the variation of the signal inherent to mass spectrometry, and (3) the effect of the matrix on the ionization of analytes. Strict regression criteria can help prevent such problems (high R^2^, negligible intercept, and restricted abatement ranges). In addition, the use of multiple competitors per target compound provides more resilience, preventing a strong bias when determining the *k*_app,O3_. To this end, it was shown that a number of competitor compounds had incorrect *k*_app,O3_, which could be problematic if they would have been used as single competitors. Therefore, a cross check of second-order rate constants is recommended for competition kinetics experiments. A limitation for the determination of second-order rate constants was the lack of competitors with *k*_app,O3_ > ∼10^7^ M^–1^ s^–1^, which was evident for the determination of *k*_app,O3_ of thioether- and tryptophan-containing cyano-metabolites. Therefore, further studies are needed to determine second-order rate constants at the higher end of the O_3_ reactivity, even though for compounds with such high reactivities, a complete abatement is already expected at low specific O_3_ doses. Overall the proposed novel approach has many advantages as a screening tool; however, if *k*_app,O3_ needs to be determined with high precision, conventional methods focusing on individual compounds should be favored, and if possible, direct methods and not competition kinetics should be applied.

The majority of the 31 cyano-metabolites identified in *Microcystis* and *Planktothrix* had *k*_app,O3_ ≥ 10^5^ M^–1^ s^–1^, indicating they should be degraded by specific O_3_ doses typically applied in drinking water treatment.^12^ Without reactive moieties such as tryptophan, thioether, phenol, and olefin, other cyano-metabolites showed significantly lower reactivity (*k*_app,O3_ ≤ 10^3^ M^–1^ s^–1^), indicating they might only be partially degraded by O_3_. In this case, the oxidation by the secondarily formed ^•^OH may enhance their abatement significantly.^39^ Because of the high molecular weights of the detected cyano-metabolites and a low selectivity of ^•^OH, it can be assumed that the second-order rate constants for the reaction of cyano-metabolites with ^•^OH are close to diffusion control (10^10^ M^–1^ s^–1^).^40,41^ For example, MC-LR and aerucyclamide A have *k*_app,OH_ values of 1.1 × 10^10^ and 6.4 × 10^9^ M^–1^ s^–1^, respectively, at pH 7.^12,42^ Furthermore, the multicompound competition kinetics allowed us to gain insight into structural moieties that have been underrepresented in the ozonation literature thus far. This is notably exemplified in this study by the high reactivity of tryptophan- and thioether-containing cyano-metabolites compared to the literature, revealing a lack of knowledge on the O_3_ reactivity of these moieties. In addition, preliminary insights were obtained on the reactivity of O_3_ with the heterocycles oxazole, thiazole, oxazoline, and thiazoline.

## Supporting Information Available

Additional experimental details, information on competitors, structures of all identified cyano-metabolites, details on *k*_O3,DMP_ redetermination, supplementary cyano-metabolite rate constants, and linear regression statistical parameters (PDF)
Spreadsheet containing the confirmation of cyano-metabolite structures by MS^2^ spectra annotation (XLSX)
