# Supplementary material for: Reactivity of Cyanobacteria Metabolites with Ozone: Multicompound Competition Kinetics
Source: Environ Sci Technol. 2024 Jun 17;58(26):11802–11. doi: 10.1021/acs.est.4c02242 (PMC11223487; doi:10.1021/acs.est.4c02242)
Supplement: Supplementary file 1 — es4c02242_si_001.pdf [file es4c02242_si_001.pdf]

SUPPORTING INFORMATION

Reactivity of cyanobacteria metabolites with ozone: Multi-compound competition kinetics

Valentin Rougé,[†] *Urs von Gunten,*[*,†,‡] *Elisabeth M.L. Janssen*[*,†]

† Eawag, Swiss Federal Institute of Aquatic Science and Technology, 8600 Dübendorf, Switzerland

‡ School of Architecture, Civil and Environmental Engineering (ENAC), École Polytechnique Fédérale de Lausanne (EPFL), 1015, Lausanne, Switzerland

* (U.v.G.) phone: +41 58 765 5270, e-mail: urs.vongunten@eawag.ch; (E.M.-L.J) phone: +41 58 765 5428, e-mail: elisabeth.janssen@eawag.ch

This PDF includes:

5 texts, 18 tables and 8 figures in 42 pages providing additional experimental details, information on competitors, structures of all identified cyano-metabolites, details on $k_{O3,DMP}$ redetermination, supplementary cyano-metabolite rate constants and linear regression statistical parameters

Table of contents

Texts

Text S1. Culture and extraction of cyanobacterial strains **S5**

Text S2. Cyano-metabolite analysis and identification **S8**

Text S3. Structure identification of cyano-metabolites by MS2 annotation **S9**

Text S4. Determination of k_{DMP} **S32**

Text S5. Estimation of cyanopeptolin B and C formation during ozonation experiments **S38**

Tables

Table S1. Reagent suppliers and purity **S4**

Table S2. Composition of WC growth medium **S6**

Table S3. List of 16 selected competitors with their species-specific k_{O3} **S12**

Table S4. List of cyanopeptides identified in *Planktothrix* and *Microcystis* extracts **S15**

Table S5. Number of data points for linear regressions at pH 7 .... **S17**

Table S6. Slope to intercept ratios of linear regressions at pH 7 .... **S19**

Table S7. R^2 of linear regressions at pH 7 .... **S21**

Table S8. Number of data points for linear regressions at pH 8 .... **S23**

Table S9. Slope to intercept ratios of linear regressions at pH 8 .... **S24**

Table S10. R^2 of linear regressions at pH 8 .... **S25**

Table S11. Structure of anabaenopeptins **S26**

Table S12. Structure of cyanopeptolins **S27**

Table S13. Structure of cyclamides **S28**

Table S14. Structure of microcystins **S29**

Table S15. $k_{app,O3}$ of dibromomethylparaben (DMP) **S32**

Table S16. $k_{app,O3}$ at pH 7 for cyano-metabolites from *Planktothrix* and *Microcystis* **S34**

Table S17. pK_a of amine-containing compounds **S37**

Table S18. $k_{app,O3}$ at pH 8 for cyano-metabolites from *Planktothrix* **S39**

Figures

Figure S1a. Stability of cyano-metabolites from *Planktothrix* **S7**

Figure S1b. Effect of blowdown method on cyano-metabolite recovery **S7**

Figure S2. Relative area variation for alachlor, diazepam and picloram.. .... **S9**

Figure S3. Screenshot of a MS2 annotation tab for one cyano-metabolite. **S11**

Figure S4. Examples of correlation plots between the abatement of cyano-metabolites and the abatement of competitors **S14**

Figure S5. Structure of unclassified cyano-metabolites **S30**

Figure S6. Stoichiometry between O_3 and carbofuran **S31**

Figure S7. Adjusted $k_{measured}$ / $k_{literature}$ of competitors in the presence of the cyano-metabolite mixture from *Planktothrix* **S33**

Figure S8. Abatement of cyanopeptolin D, C and B from *Microcystis* at pH 7 .... **S37**

Table S1. Reagent suppliers and purity

Chemical	Supplier, purity	Chemical	Supplier, purity
Acetyl-sulfamethoxazole	abcr, 96%	$NaH_2PO_4.H_2O$	Sigma Aldrich, ≥ 99%
Alachlor	HPC Standards Gmbh, 99%	$Na_2HPO_4.2H_2O$	Sigma Aldrich, ≥ 98%
Bezafibrate	TCI Europe, 98%	Cinnamic acid	Sigma Aldrich, ≥ 99%
Carbamazepine	Sigma Aldrich, ≥ 98%	Phenol	Sigma Aldrich, 99.5%
Carbofuran	Pestanal, 99%	Formic acid	Sigma Aldrich, ≥ 98%
Ciprofloxacin HCl	Supelco, 99%	Methanol	Fischer scientific, Optima LC-MS grade
Diazepam	Lipomed AG, 98.5%	Acetone	Supelco, ≥ 99.8%
Methyl 3,5-dibromo-4-hydroxybenzoate	Alfa Aesar, 98%	Tert-butanol	Sigma Aldrich, ≥ 99.7%
Penicillin G sodium salt	Sigma Aldrich, 96%	Microcystin-LR	Enzo life science, >95% (HPLC area)
Picloram	Sigma Aldrich, 98.2%	[D-Asp3]Microcystin-LR	Enzo life science, >95% (HPLC area)
Roxithromycin	TRC-Canada, 98%	[D-Asp3,(E)-Dhb7]Microcystin-RR	CyanoBiotech GmbH, >95% (HPLC area)
Sulfamethoxazole	Sigmal Aldrich, 99.6%	Cyanopeptolin A	CyanoBiotech GmbH, >90% (HPLC area)
Tramadol HCl	Sigmal Aldrich, 99.9%	Cyanopeptolin D	CyanoBiotech GmbH, >90% (HPLC area)
Triclosan	HPC Standards Gmbh, 99.9%	Anabaenopeptin A	CyanoBiotech GmbH, >90% (HPLC area)
Trimethoprim	Sigma Aldrich, 98.5%	Anabaenopeptin B	CyanoBiotech GmbH, >90% (HPLC area)
Tylosin tartrate	Fluka, 90%	Oscillamide Y	CyanoBiotech GmbH, >90% (HPLC area)
Vancomycin HCl	TRC-Canada, 96.2%		

Text S1. Culture and extraction of cyanobacterial strains

Microcystis aeruginosa was originally isolated from Braakman reservoir in the Netherlands and was obtained from the Pasteur Culture Collection of Cyanobacteria (France, strain PCC7806). *Planktothrix rubescens* was originally isolated from Lake Borre Sø in Denmark and obtained from the Norwegian Culture Collection of Algae (NORCCA, strain K-0576). Primary cultures were kept in 75-mL modified WC medium (Table S2) at 20 ± 2 °C and irradiated at 12 µmol photons m^{-2} s^{-1} on a 12:12-h light/dark cycle.[1] To produce a significant amount of biomass, both strains were grown in 4.5 L inoculated with 10-15% inoculum in 5-L Schott bottles as described above and aerated with filtered air (GE Healthcare, Whatman, HEPA-VENT, 0.3 µm). All materials used for culturing were autoclaved before use and all the subculturing was performed under sterile conditions. The cells were harvested by centrifugation (rcf of 4000 g at 10 °C, 10 min, Herolab HiCen XL), lyophilized (-40 °C, -3 mbar, 24 h, Lyovac GT2, Leybold) and stored at -20 °C until further use.[2] To extract cyano-metabolites, 16 mL MeOH/H_2O (70/30 v/v) was added to 200 mg of the dried harvested cells. The suspension was homogenized by vortex, sonicated (level 9, 10 min at 15 °C, Ultrasonic cleaner USC-THD, VWR) and centrifugated (5 min at 2600 rpm and 10 °C, Megafuge 1.0 R, Thermo Scientific). The supernatant was withdrawn and the extraction repeated a second time. Supernatants were then combined and diluted by about a factor of 30 in pure water (final volume 1L). The solution was then split in two and each fraction was loaded on a solid-phase extraction (SPE) cartridge (HLB 6cc, 200mg, Oasis) preconditioned by MeOH followed by pure water. Cartridges were then washed by 9 mL pure water and 9 mL MeOH/H_2O (20/80 v/v), and eluted by 9 mL MeOH/H_2O (85/15 v/v). The extracts could be conserved in this mixture at -20°C for several months without degradation of the identified cyano-metabolites (Figure S1a).

Table S2. Composition of WC growth medium (modified from Guillard and Lorenzen, 1972).[1]

Components	Concentration (mg L^{-1})
$K_2HPO_4 \cdot 3H_2O$	11.4
$NaNO_3$	85
$CaCl2 \cdot 2H_2O$	36.8
$MgSO_4 \cdot 7H_2O$	37
$NaHCO_3$	12.6
Na_2EDTA	4.36
$FeCl_3 \cdot 6H_2O$	3.15
$CuSO_4 \cdot 5H_2O$	0.01
$ZnSO_4 \cdot 7H_2O$	0.022
$CoCl_2 \cdot 6H_2O$	0.01
$MnCl_2 \cdot 4H_2O$	0.18
$Na_2MoO4 \cdot 2H_2O$	0.006
H_3BO_3	1.00
N-[Tris(hydroxymethyl)methyl]-2-aminoethanesulfonic acid sodium salt (TES) buffer	115

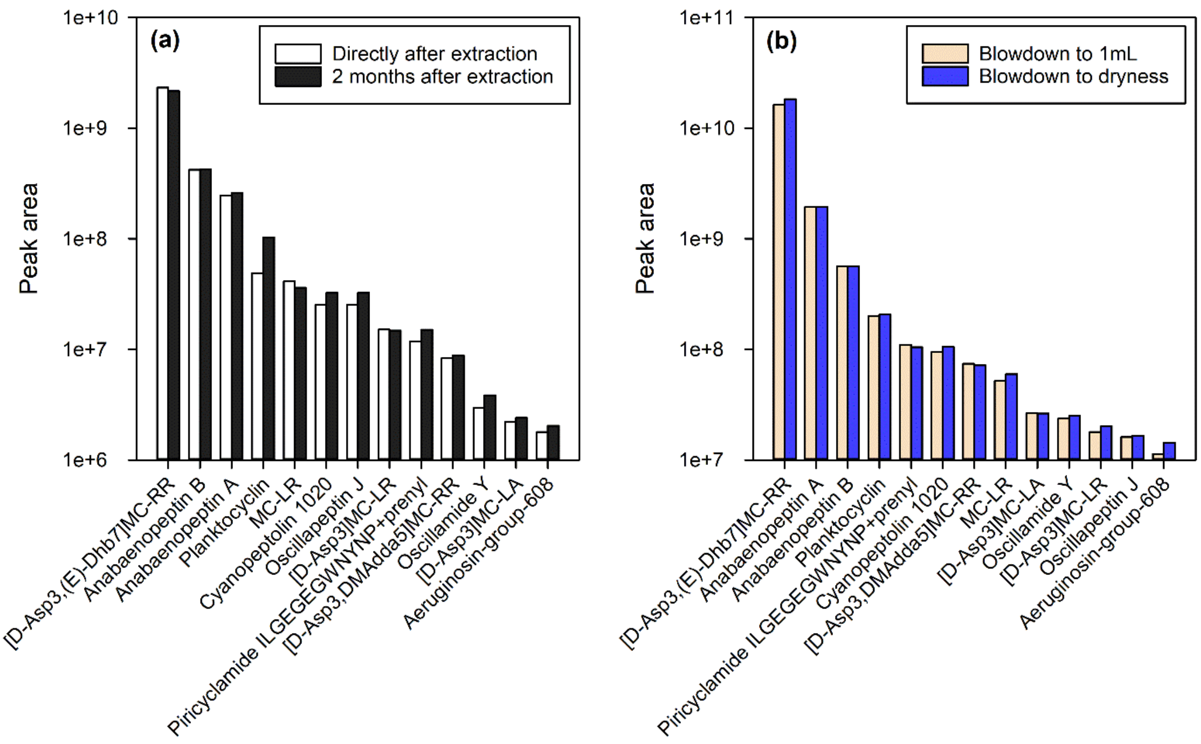

Figure S1. (a) Stability of cyano-metabolites from a *Planktothrix* extract when kept at -20°C in 85/15 v/v MeOH/water. The peak areas of cyano-metabolites in the same extract were measured just after extraction (white bars) and after 2 months (black bars). (b) Effect of blowdown method of *Planktothrix* extract on cyano-metabolite recovery. 30 mL of diluted extract was blown down to 1 mL (orange bars), or to dryness (blue bars) before being diluted/redissolved to the same volume.

Text S2. Cyano-metabolite analysis and identification

Cyano-metabolites were analyzed by HPLC (Dionex Ultimate 3000 RS pump, Thermo Fischer Scientific) with a Kinetex C18 column (2.6µm, 2.1×100 mm, Phenomenex, precolumn VanGuard, Cartridge, Waters), coupled to a high-resolution tandem mass spectrometer (HRMS/MS, Exploris, ThermoFisher Scientific). [3, 4] 20 µL were injected and eluted using nanopure water and MeOH, both acidified with formic acid (0.1%). A gradient elution was carried out at a flowrate of 0.255mL min^{-1}: MeOH at 7% for 3 min, increasing to 30% between 3 and 7 min, to 70% between 7 and 30 min, and to 100% between 30 and 32 min. HRMS/MS used electrospray ionization (ESI) with 320°C capillary temperature, and both positive and negative ionization modes with 3.5 kV and 2.5 kV electrospray voltage, respectively. Full scan from 150 to 1500 m/z was used with a nominal resolution of 120000 at m/z 250, 1×10^6 automated gain control (AGC), 100 ms maximal injection time, and 1 ppm mass accuracy. Internal mass calibration was done on each run start (EASY-IC) to prevent mass drift overtime. High-resolution product ion spectra were obtained by normalized collision energies for HCD of 15%, 30%, 45% at a resolving power of 15000 at 400m/z, 5×10^4 AGC, 70 ms maximal injection time, 1 m/z isolation window, triggering data-dependent MS/MS acquisition using compounds in CyanoMetDB (version 01, April 2021). For the monitoring of cyano-metabolites during ozonation experiments, only HCD of 30% was kept, leading to a better definition of the peaks. Skyline 22.2 (MacCoss LabSoftware) was used for the integration of the peaks. MS1 filtering settings were the same as the measurement settings, i.e, m/z scan range between 150 and 1500, and resolving power of 120000 at m/z 250. The cumulated integration of the peak areas of up to M+3 isotopes was used. Minimal matrix effects were observed for the three least reactive competitors diazepam, picloram and alachlor, for which no reaction with O_3 was expected at the O_3 doses used (Figure S2). More than 90% of peak areas did not vary by more than ± 10%. In addition, the cause of most of outliers was identified to be a dilution error during O_3 spiking, and diazepam, picloram and alachlor could be used to correct all other compounds.

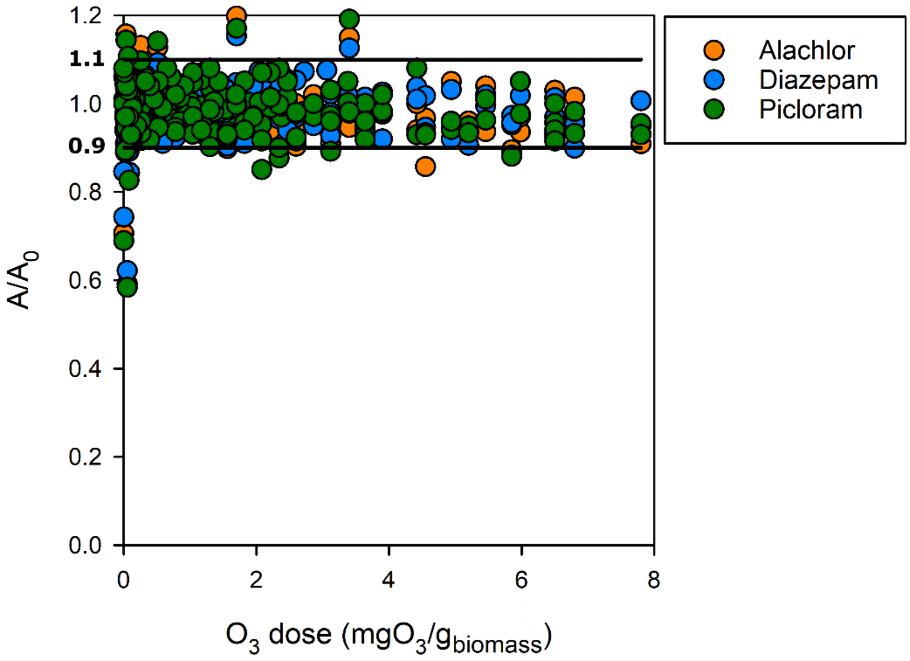

Figure S2. Relative area variation for the three unreactive competitors alachlor, diazepam and picloram. All the data points from experiments with both Planktothrix and Microcystis are shown. The lines represent a relative variation of ±10%.

Text S3. Structure identification of cyano-metabolites by MS^2 annotation

The details of the MS^2 annotation of cyano-metabolites are provided in a separate spreadsheet supporting information (SI2). Each cyano-metabolite MS^2 annotation is provided in an individual tab. An example is shown in Figure S3. The different sections of Figure S3 are explained in the following.

The name of the cyano-metabolite and the sample in which it was analyzed for MS^2 annotation are shown in part A in Figure S3. MS^2 spectra at two or three different collision energies (HCD) are shown merged in part B, with relevant peaks numbered. The full list of peaks is provided in part C and the list of relevant numbered peaks is given in part D. The

relevant peaks are a representative selection of peaks that can explain the overall structure of the cyano-metabolite, which are not necessarily the most intense ones. A more exhaustive annotation was done for all cyano-metabolites but is not shown here.

The details of each fragment associated with each relevant peak is provided in part E. Details include molecular formula, measured and theoretical m/z, mass error, structure and fragment name. Most of the fragment structures were created by Metfrag web,[5] and were shown as highlighted in green over the full structure. For the naming of fragments, amino acids are named using their official abbreviation and separated by vertical bars representing peptide bonds (e.g., [z/Dha|Ala|Arg|DAsp|Arg/c]+H^+). For non-standard amino acids, their full name and, if relevant, their SMILES code are given in a tab "Abbreviations". Forward slashes associated with a letter (a, b, c, x, y or z) are used to represent the fragmentation site and the corresponding type of peptide bond fragmentation.[6] Small losses, such as hydrogen, water or ammonia loss, at unspecified sites are indicated at the end of the fragment (e.g., [z/Ahp|Leu|NMePhe/c-H_2O]+H^+). Other specific losses/fragmentations are written in italic (e.g., [x/MeOxazoline|Gly|Thiazole|Met*(minus* SCH_3*)*|Thiazoline/b]$^+$).

Key points of the interpretation of the MS^2 fragments are provided in part F, notably explaining how the different selected fragments can explain the overall structure. Comparison with other similar cyano-metabolites, notably those annotated from bioreagents, was often done to further confirm the structures. For cyano-metabolites annotated from bioreagents, comparison of the MS^2 spectra between the bioreagent and the cyanobacteria extract is shown in a tab "Head-to-tail plots".

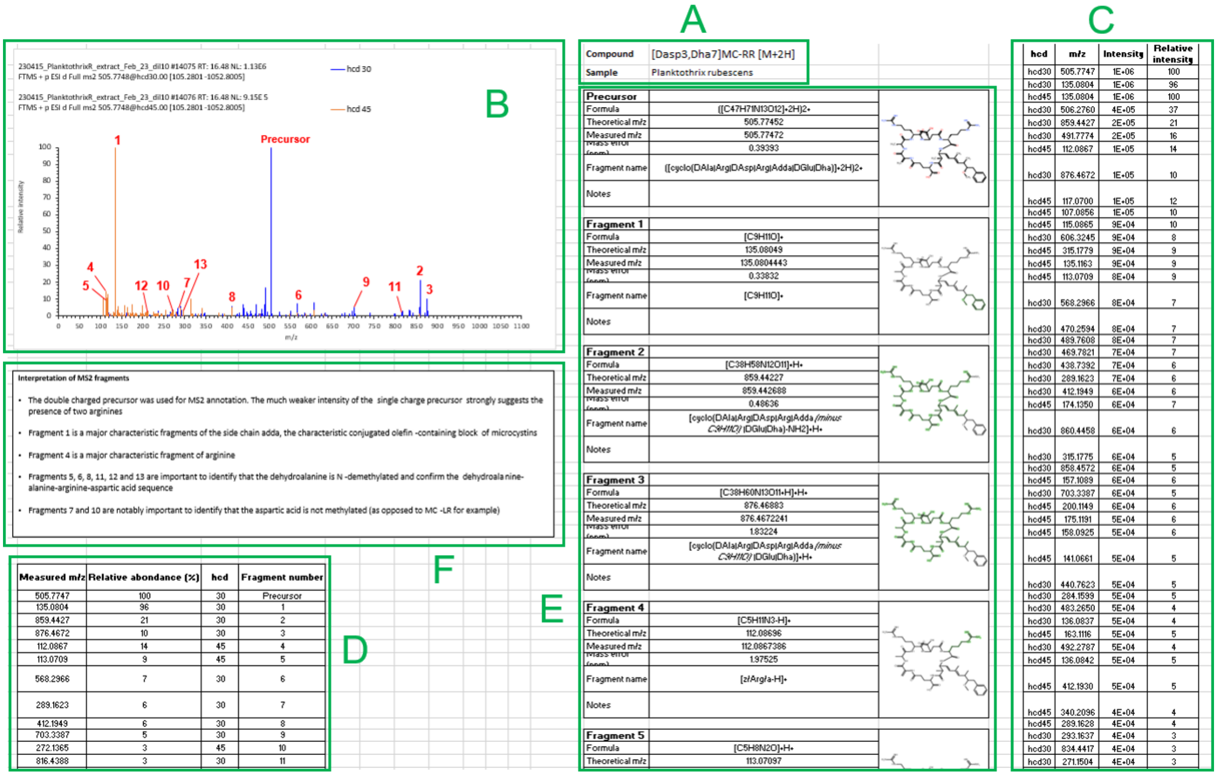

Figure S3. Screenshot of a MS2 annotation tab for one cyano-metabolite. See Text S3 for explanations of the parts A-C.

Table S3. List of 16 selected competitors including their chemical structures and the corresponding species-specific second-order rate constants for their reactions with O_3.

Competitor (abbreviation)	Structure	pK_a [b]	k_{O3} (M^{-1} s^{-1}) [c]	Retention time (min)
Acetyl-sulfamethoxazole (ASMX)	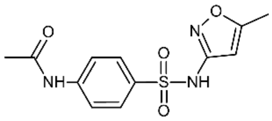	5.5	$k_1 = (2.0 \pm 0.2) \times 10^{1}$ [7]<br>$k_2 = (2.6 \pm 0.1) \times 10^{2}$ [7]	10.1
Alachlor (ALA)	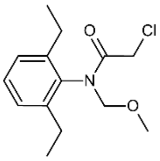	N/A	3.8 ± 0.4 [8]	25.5
Bezafibrate (BZF)	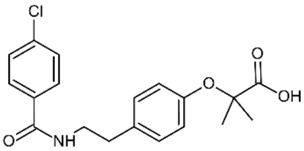	N/A	$(5.9 \pm 0.5) \times 10^{2}$ [9]	23.9
Carbamazepine (CBZ)	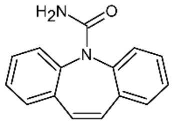	N/A	$(6.1 \pm 0.1) \times 10^{5}$ [10]	16.4
Carbofuran (CBF)	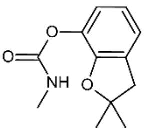	N/A	6.2×10^{2} [8]<br>2.1×10^{2} [d]	14.3
Ciprofloxacin (CF)	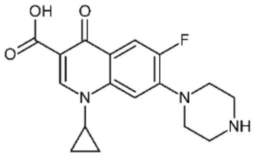	6.2, 8.8	$k_1 = (4.0 \pm 1.2) \times 10^{2}$ [7]<br>$k_2 = (7.5 \pm 2.8) \times 10^{3}$ [7]<br>$k_3 = (9.0 \pm 3.1) \times 10^{5}$ [7]	7.6
Diazepam (DZP)	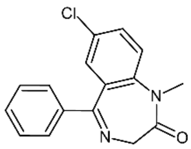	N/A	$(7.5 \pm 0.15) \times 10^{-1}$ [9]	21.8
Dibromo-methylparaben [a] (DMP)	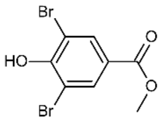	4.7	$k_1 = 2.2 \times 10^{1}$ [11]<br>$k_2 = 8.4 \times 10^{7}$ [11]<br>$k_2 = (4.3 \pm 0.3) \times 10^{6}$ [e]	21.8
Penicillin G (PG)	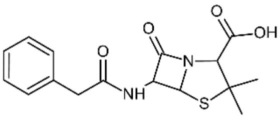	N/A	$(4.8 \pm 0.1) \times 10^{3}$ [7]	16.0

Table S3 continued.

Picloram (PCL)	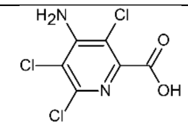	3.6	$k_1 = (5.0 \pm 1.0) \times 10^{1}$ [8]<br>$k_2 = (1.4 \pm 0.2) \times 10^{2}$ [8]	7.5
Roxithromycin (ROX)	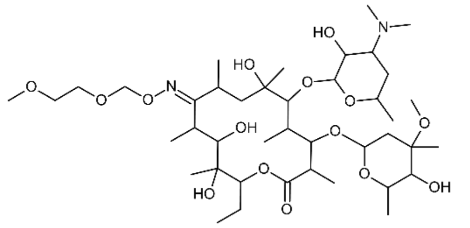	9.2	$k_1 < 1$ [7]<br>$k_2 = (1.0 \pm 0.1) \times 10^{7}$ [7]	22.0
Sulfamethoxazole (SMX)	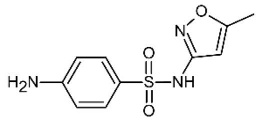	5.6	$k_1 = (9.4 \pm 1.8) \times 10^{4}$ [f,7]<br>$k_2 = (1.1 \pm 0.2) \times 10^{6}$ [f,7]	8.0
Tramadol (TRA)	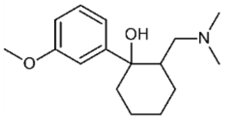	9.4	$k_1 = (7.7 \pm 0.2) \times 10^{1}$ [12]<br>$k_2 = (1.0 \pm 0.1) \times 10^{6}$ [12]	7.9
Triclosan (TRI)	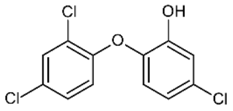	8.1	$k_1 = (1.3 \pm 0.1) \times 10^{3}$ [13]<br>$k_2 = (5.1 \pm 0.1) \times 10^{8}$ [13]	31.4
Trimethoprim (TMP)	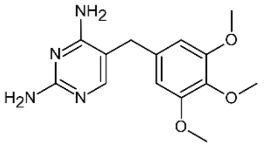	3.2, 7.1	$k_1 = (6.6 \pm 6) \times 10^{4}$ [f,7]<br>$k_2 = (1.5 \pm 0.4) \times 10^{5}$ [f,7]<br>$k_3 = (1.0 \pm 0.2) \times 10^{6}$ [f,7]	6.6
Tylosin (TYL)	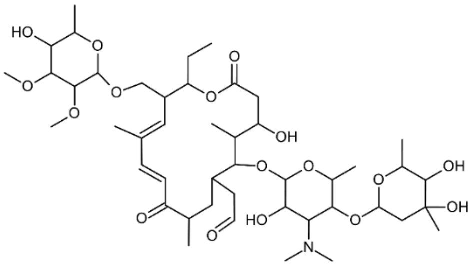	7.7	$k_1 = (1.5 \pm 0.3) \times 10^{5}$ [f,7]<br>$k_2 = (5.4 \pm 1.0) \times 10^{6}$ [f,7]	18.5

[a] IUPAC name: methyl 3,5-dibromo-4-hydroxybenzoate

[b] Only pK_a affecting reactivity in the pH range 4-12 are shown. N/A indicates that the compound doesn't have a pK_a or that it doesn't impact the reactivity.

[c] k_1, k_2 and k_3 refer to the species-specific second-order rate constants from the most protonated to the most deprotonated species of the competitor, whenever it applies.

[d] Second-order rate constant corrected for stoichiometry. See explanations in Section "Validation of competitors" in the main manuscript.

[e] Second-order rate constants re-determined using cinnamic acid and phenol as competitors. See details in Section "Validation of competitors" in the main manuscript and Text S4.

[f] Second-order rate constants recalculated based on the re-evaluated second-order rate constant for cinnamic acid by Kim et al. 2019 [14]

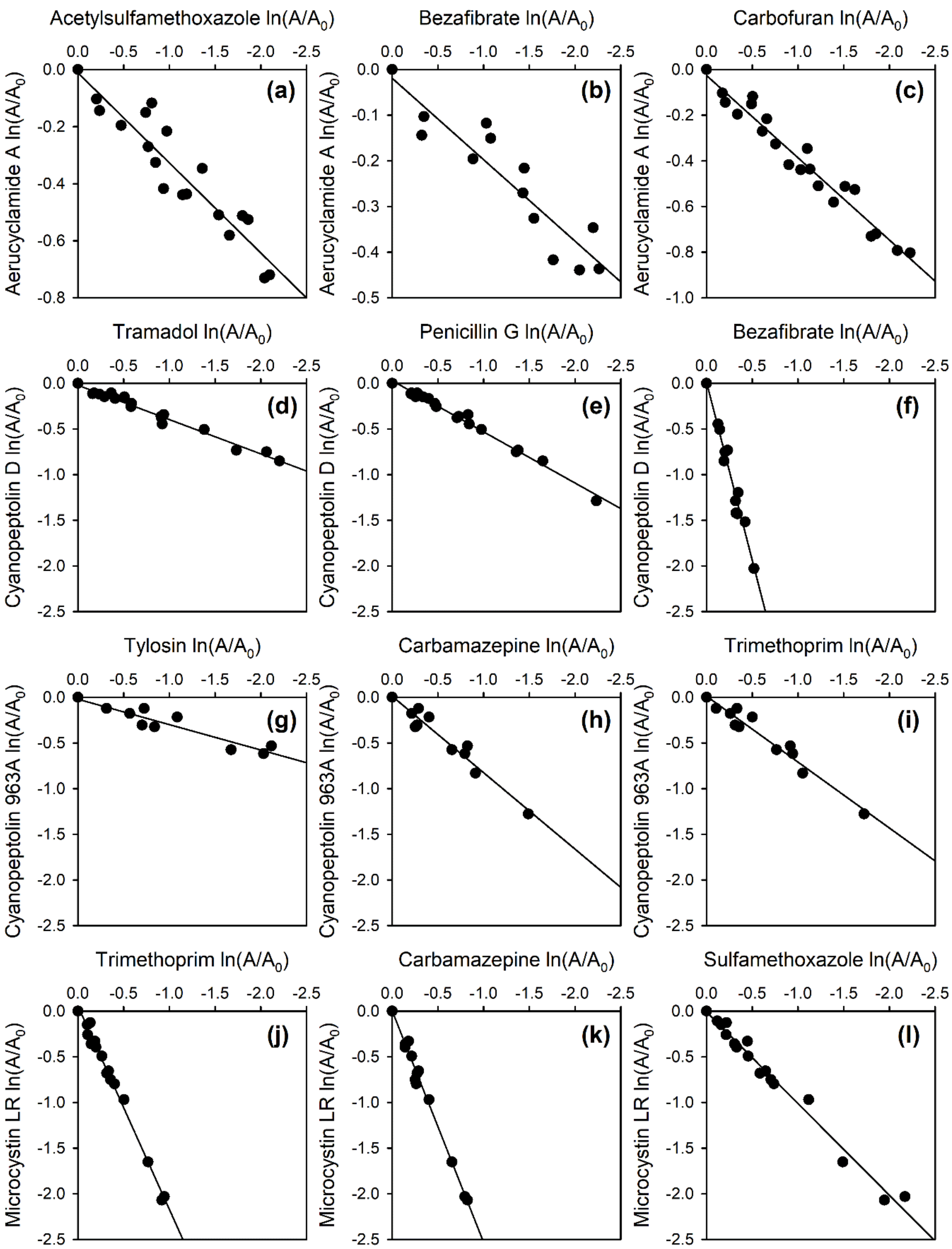

Figure S4. Examples of correlation plots between the ln of the relative residual peaks of cyano-metabolites and competitors upon ozonation of the cyano-metabolite mixture from 0.6 $g_{biomass}$ L^{-1} of *Microcystis* at pH 7 (2 mM phosphate), 22°C, and in presence of *tert*-butanol (40 mM). The correlation plots between (a-c) aerucyclamide A, (d-f) cyanopeptolin D, (g-i) cyanopeptolin 963A and (j-l) microcystins LR and three corresponding competitors are shown.

Table S4. List of cyanopeptides identified in *Planktothrix* and *Microcystis* extracts. The structures are shown in Tables S11-S14 and Figure S5.

Compound Name	CyanometDB ID #	Cyanobacterial strain	Molecular Formula	Retention time (min)	Confidence level of identification[15]	Ion precursors monitored
**Aeruginosin**						
Aeruginosin-group-608[a]	1198, 1199, 1200	*Planktothrix*	$C_{32}H_{44}N_6O_6$	10.8	2b-3	$[M+H]^+$
**Anabaenopeptin**						
Anabaenopeptin A	760	*Planktothrix*	$C_{44}H_{57}N_7O_{10}$	21.3	1	$[M+H]^+$, $[M-H]^-$
Anabaenopeptin B	867	*Planktothrix*	$C_{41}H_{60}N_{10}O_9$	14.2	1	$[M+H]^+$, $[M+2H]^{2+}$
Anabaenopeptin F	805	*Planktothrix*	$C_{42}H_{62}N_{10}O_9$	15.8	2b-3	$[M+H]^+$, $[M+2H]^{2+}$
Anabaenopeptin SA13	2096	*Planktothrix*	$C_{44}H_{57}N_7O_{11}$	17.8	2b-3	$[M+H]^+$, $[M-H]^-$
Oscillamide Y	713	*Planktothrix*	$C_{45}H_{59}N_7O_{10}$	22.8	1	$[M+H]^+$, $[M-H]^-$
**Cyanopeptolin**						
Cyanopeptolin 1020	512	*Planktothrix*	$C_{50}H_{72}N_{10}O_{13}$	20.6	2b-3	$[M+H]^+$, $[M-H]^-$
Cyanopeptolin 963A	555	*Microcystis*	$C_{49}H_{69}N_7O_{13}$	30.0	2b-3	$[M+H]^+$, $[M-H]^-$
Cyanopeptolin A	640	*Microcystis*	$C_{46}H_{72}N_{10}O_{12}$	24.9	1	$[M+H]^+$, $[M-H]^-$
Cyanopeptolin B	642	*Microcystis*	$C_{46}H_{72}N_8O_{12}$	24.9	2b-3	$[M+H]^+$, $[M-H]^-$
Cyanopeptolin C	603	*Microcystis*	$C_{47}H_{74}N_8O_{12}$	24.9	2b-3	$[M+H]^+$, $[M-H]^-$
Cyanopeptolin D	571	*Microcystis*	$C_{48}H_{76}N_8O_{12}$	24.9	1	$[M+H]^+$, $[M-H]^-$
Oscillapeptin J	613	*Planktothrix*	$C_{47}H_{68}N_{10}O_{18}S$	15.2	2b-3	$[M+H]^+$, $[M-H]^-$
**Cyclamide**						
Aerucyclamide A	1466	*Microcystis*	$C_{24}H_{34}N_6O_4S_2$	28.5	1	$[M+H]^+$
Aerucyclamide B	1472	*Microcystis*	$C_{24}H_{32}N_6O_4S_2$	30.3	2b-3	$[M+H]^+$
Aerucyclamide C	1469	*Microcystis*	$C_{24}H_{32}N_6O_5S$	29.9	2b-3	$[M+H]^+$

Table S4 continued.

Aerucyclamide D	1402	*Microcystis*	$C_{26}H_{30}N_6O_4S_3$	25.9	2b-3	$[M+H]^+$
Microcyclamide 7806A[c]	NA[c]	*Microcystis*	$C_{24}H_{34}N_6O_6S$	17.7	2b-3	$[M+H]^+$, $[M-H]^-$
Microcyclamide 7806B	1465	*Microcystis*	$C_{24}H_{34}N_6O_6S$	22.0	2b-3	$[M+H]^+$, $[M-H]^-$
**Microcystin**						
Microcystin-group-967[b]	1806, 1807	*Microcystis*	$C_{47}H_{70}N_{10}O_{12}$		3	
[D-Asp^3,(E)-Dhb^7]MC-RR	1962	*Planktothrix*	$C_{48}H_{73}N_{13}O_{12}$	16.7	1	$[M+2H]^{2+}$, $[M-H]^-$, $[M-2H]^{2-}$
[D-Asp^3,Dha^7]MC-RR	1884	*Planktothrix*	$C_{47}H_{71}N_{13}O_{12}$	16.3	2b-3	$[M+H]^+$, $[M+2H]^{2+}$, $[M-H]^-$
[D-Asp^3,DMAdda^5]MC-RR	1885	*Planktothrix*	$C_{47}H_{71}N_{13}O_{12}$	12.2	2b-3	$[M+2H]^{2+}$
[D-Asp^3]MC-LA	1785	*Planktothrix*	$C_{45}H_{65}N_7O_{12}$	28.4	1	$[M+H]^+$, $[M-H]^-$
[D-Asp^3]MC-LR	1953	*Microcystis and Planktothrix*	$C_{48}H_{72}N_{10}O_{12}$	23.1	1	$[M+H]^+$, $[M+2H]^{2+}$, $[M-H]^-$
[Mdha-GSH^7]MC-LR	2071	*Microcystis*	$C_{59}H_{91}N_{13}O_{18}S$	20.2	3	$[M+2H]^{2+}$, $[M-2H]^{2-}$
MC-HilR	1879	*Microcystis*	$C_{50}H_{76}N_{10}O_{12}$	24.1	2b-3	$[M+2H]^{2+}$, $[M-H]^-$
MC-LAba	1790	*Planktothrix*	$C_{47}H_{69}N_7O_{12}$	30.2	2b-3	$[M+H]^+$, $[M-H]^-$
MC-LR	1823	*Microcystis and Planktothrix*	$C_{49}H_{74}N_{10}O_{12}$	22.7	1	$[M+H]^+$, $[M+2H]^{2+}$, $[M-H]^-$
**Other**						
Piricyclamide ILGEGEGWNYNP+prenyl	107	*Planktothrix*	$C_{66}H_{91}N_{15}O_{19}$	27.2	3	$[M+2H]^{2+}$, $[M-2H]^{2-}$
Planktocyclin	939	*Planktothrix*	$C_{39}H_{60}N_8O_8S$	28.2	2b-3	$[M+H]^+$, $[M-H]^-$

[a] Refers to either of the stereoisomer Microcin SF608, Aeruginosin KT608A and Aeruginosin KT608B

[b] Refers to either of the isobar [D-Asp3,DMAdda5,Dhb7]MC-LR or [D-Asp3,DMAdda5]MC-LR

[c] Refers to the structure corrected by Portmann et al.[16, 17] In the CyanoMetDB version used (v02, 2023), the entry for Microcyclamide 7806A was the previously proposed structure by Ziemert et al.[18]

Table S5. Number of data points for linear regressions at pH 7.

Cyano-metabolites \ Competitors	ASMX	BZF	CBF	TRA	PG	CIP	ROX	TMP	CBZ	TYL	SMX	DMP	TRI
**Tryptophan**													
Piricyclamide ILGEGEGWNYNP+prenyl													14
**Thioethers**													
[Mdha-GSH7]MC-LR								16		16	16	16	
Aerucyclamide D												12	10
Planktocyclin												10	17
**Olefins**													
[D-Asp3,(E)-Dhb7]MC-RR								15	15	19	19	10	
[D-Asp3,Dha7]MC-RR								15	15	19	20	10	
[D-Asp3,DMAdda5]MC-RR								13	13	17	18	10	
[D-Asp3]MC-LA								16	16	19	20		
[D-Asp3]MC-LR (Microcystis)								17	14	17	17	11	
[D-Asp3]MC-LR (Planktothrix)								15	15	18	20		
MC-HilR								19	14	17	18	13	
MC-LAba								15	15	20	21	10	
MC-LR (Microcystis)								17	14	18	18	11	
MC-LR (Planktothrix)								16	16	18	20	11	
Microcystin-group-967								17	13	16	15	12	
**Phenols**													
Aeruginosin-group-608								13	13	17	19	10	
Anabaenopeptin A								16	16	20	23	10	
Anabaenopeptin B								17	17	20	20		
Anabaenopeptin F								16	16	19	19		
Anabaenopeptin SA13								15	15	18	19	10	

Table S5 continued.

Cyanopeptolin 1020								17	17	19	21		
Cyanopeptolin 963A [a]								14	13	13	13		
Oscillamide Y								15	15	18	18		
Oscillapeptin J								13	13	17	19	10	
**Amines**													
Cyanopeptolin B [a]													
Cyanopeptolin C [a]	23	17	24										
Cyanopeptolin D [a]	10	13	10	18	21								
**Heterocycles**													
Aerucyclamide A	20	16	21										
Aerucyclamide B													
Aerucyclamide C		10		17	19								
Microcyclamide 7806A		10		17	20								
Microcyclamide 7806B		10		17	21								

Table S6. Slope to intercept ratios of linear regressions at pH 7.

Competitors / Cyano-metabolites	ASMX	BZF	CBF	TRA	PG	CIP	ROX	TMP	CBZ	TYL	SMX	DMP	TRI
**Tryptophan**													
Piricyclamide ILGEGEGWNYNP+prenyl													-10
**Thioethers**													
[Mdha-GSH7]MC-LR								130		-24	-41	-12	
Aerucyclamide D												-1038	954
Planktocyclin												-282	-18
**Olefins**													
[D-Asp3,(E)-Dhb7]MC-RR								-16	-29	30	45	266	
[D-Asp3,Dha7]MC-RR								-14	-19	436	321	23	
[D-Asp3,DMAdda5]MC-RR								-19	-49	205	27	10	
[D-Asp3]MC-LA								-19	-26	717	-278		
[D-Asp3]MC-LR (Microcystis)								33	123	21	42	14	
[D-Asp3]MC-LR (Planktothrix)								-36	-60	17	19		
MC-HilR								36	88	78	-90	-10	
MC-LAba								-17	-36	53	54	18	
MC-LR (Microcystis)								42	420	32	156	29	
MC-LR (Planktothrix)								-16	-28	4823	-184	-36	
Microcystin-group-967								92	-86	-34	-14	-15	
**Phenols**													
Aeruginosin-group-608								-24	-97	138	25	28	
Anabaenopeptin A								-15	-22	-68	-49	38	
Anabaenopeptin B								-14	-23	149	-920	59	
Anabaenopeptin F								-14	-22	-92	-54		
Anabaenopeptin SA13								-16	-24	35	-2294	452	

Table S6 continued.

Cyanopeptolin 1020								-13	-19	-22	-21		
Cyanopeptolin 963A [a]								223	-12	-15	-10		
Oscillamide Y								-24	-85	28	52		
Oscillapeptin J								-18	-40	-131	33	12	
**Amines**													
Cyanopeptolin B [a]													
Cyanopeptolin C [a]	-160	-10	66										
Cyanopeptolin D [a]	-73	-23	690	-12	121								
**Heterocycles**													
Aerucyclamide A	51	41	51										
Aerucyclamide B													
Aerucyclamide C		196		-56	29								
Microcyclamide 7806A		103		-166	15								
Microcyclamide 7806B		-1835		-11	-170								

Table S7. R^2 of linear regressions at pH 7.

Competitors / Cyano-metabolites	ASMX	BZF	CBF	TRA	PG	CIP	ROX	TMP	CBZ	TYL	SMX	DMP	TRI
**Tryptophan**													
Piricyclamide ILGEGEGWNYNP+prenyl													0.917
**Thioethers**													
[Mdha-GSH7]MC-LR								0.967		0.990	0.967	0.985	
Aerucyclamide D												0.937	0.959
Planktocyclin												0.907	0.923
**Olefins**													
[D-Asp3,(E)-Dhb7]MC-RR								0.973	0.958	0.996	0.991	0.946	
[D-Asp3,Dha7]MC-RR								0.965	0.950	0.985	0.991	0.961	
[D-Asp3,DMAdda5]MC-RR								0.943	0.930	0.990	0.992	0.959	
[D-Asp3]MC-LA								0.969	0.956	0.989	0.993		
[D-Asp3]MC-LR (Microcystis)								0.993	0.991	0.994	0.990	0.915	
[D-Asp3]MC-LR (Planktothrix)								0.966	0.970	0.950	0.981		
MC-HilR								0.987	0.971	0.984	0.984	0.910	
MC-LAba								0.953	0.923	0.973	0.974	0.936	
MC-LR (Microcystis)								0.993	0.992	0.996	0.981	0.942	
MC-LR (Planktothrix)								0.978	0.971	0.987	0.995	0.954	
Microcystin-group-967								0.967	0.939	0.981	0.954	0.902	
**Phenols**													
Aeruginosin-group-608								0.958	0.933	0.984	0.983	0.985	
Anabaenopeptin A								0.990	0.983	0.994	0.993	0.997	
Anabaenopeptin B								0.988	0.980	0.998	0.993		
Anabaenopeptin F								0.964	0.954	0.992	0.987		
Anabaenopeptin SA13								0.929	0.902	0.978	0.961	0.984	

Table S7 continued.

Cyanopeptolin 1020								0.986	0.984	0.947	0.950		
Cyanopeptolin 963A [a]								0.979	0.969	0.911	0.903		
Oscillamide Y								0.972	0.958	0.983	0.992		
Oscillapeptin J								0.952	0.927	0.993	0.992	0.947	
**Amines**													
Cyanopeptolin B [a]													
Cyanopeptolin C [a]	0.933	0.901	0.952										
Cyanopeptolin D [a]	0.933	0.943	0.934	0.972	0.982								
**Heterocycles**													
Aerucyclamide A	0.955	0.908	0.955										
Aerucyclamide B													
Aerucyclamide C		0.958		0.988	0.994								
Microcyclamide 7806A		0.950		0.986	0.994								
Microcyclamide 7806B		0.961		0.971	0.980								

Table S8. Number of data points for linear regressions at pH 8.

Competitors / Cyano-metabolites	ROX	TMP	CBZ	TYL	SMX	DMP	TRI
**Tryptophan**							
Piricyclamide ILGEGEGWNYNP+prenyl							14
**Thioethers**							
Planktocyclin						10	17
**Olefins**							
[D-Asp3]MC-LR		15	15	19	19	10	
[D-Asp3,(E)-Dhb7]MC-RR		15	15	19	20	10	
[D-Asp3]MC-LA		13	13	17	18	10	
[D-Asp3,Dha7]MC-RR		16	16	19	20		
[D-Asp3,DMAdda5]MC-RR		17	14	17	17	11	
MC-LR		15	15	18	20		
MC-LAba		19	14	17	18	13	
**Phenols**							
Anabaenopeptin A		13	13	17	19	10	
Anabaenopeptin B		16	16	20	23	10	
Anabaenopeptin F		17	17	20	20		
Anabaenopeptin SA13		16	16	19	19		
Cyanopeptolin 1020		15	15	18	19	10	
Aeruginosin-group-608		17	17	19	21		
Oscillamide Y		14	13	13	13		
Oscillapeptin J		15	15	18	18		

Table S9. Slope to intercept ratios of linear regressions at pH 8.

Cyano-metabolites \ Competitors	ROX	TMP	CBZ	TYL	SMX	DMP	TRI
**Tryptophan**							
Piricyclamide ILGEGEGWNYNP+prenyl						-52	14
**Thioethers**							
Planktocyclin						-33	49
**Olefins**							
[D-Asp3]MC-LR	18	66	41		24	-11	
[D-Asp3,(E)-Dhb7]MC-RR	15	864	103	-25	18	112	
[D-Asp3]MC-LA	12	-73	63		21	-10	
[D-Asp3,Dha7]MC-RR	11	-2443	106	-45	14	41	
[D-Asp3,DMAdda5]MC-RR	13	105	147	-23	17	14	
MC-LR	19	-21	115		-178	-13	
MC-LAba	10	92	30	35	11	13	
**Phenols**							
Anabaenopeptin A				-119		93	
Anabaenopeptin B				-51		67	
Anabaenopeptin F				-26		-18	
Anabaenopeptin SA13				49		24	
Cyanopeptolin 1020				-20		43	
Aeruginosin-group-608				-73		123	
Oscillamide Y				41		34	
Oscillapeptin J				-131		56	

Table S10. R^2 of linear regressions at pH 8.

Cyano-metabolites \ Competitors	ROX	TMP	CBZ	TYL	SMX	DMP	TRI
**Tryptophan**							
Piricyclamide ILGEGEGWNYNP+prenyl						0.966	0.953
**Thioethers**							
Planktocyclin						0.963	0.899
**Olefins**							
[D-Asp3]MC-LR	0.917	0.972	0.967		0.956	0.912	
[D-Asp3,(E)-Dhb7]MC-RR	0.903	0.965	0.943	0.973	0.945	0.991	
[D-Asp3]MC-LA	0.970	0.989	0.985		0.978	0.962	
[D-Asp3,Dha7]MC-RR	0.976	0.991	0.987	0.946	0.986	0.993	
[D-Asp3,DMAdda5]MC-RR	0.983	0.996	0.987	0.976	0.988	0.993	
MC-LR	0.966	0.987	0.977		0.981	0.985	
MC-LAba	0.964	0.974	0.982	0.912	0.967	0.902	
**Phenols**							
Anabaenopeptin A				0.922		0.995	
Anabaenopeptin B				0.962		0.974	
Anabaenopeptin F				0.960		0.956	
Anabaenopeptin SA13				0.949		0.993	
Cyanopeptolin 1020				0.974		0.999	
Aeruginosin-group-608				0.939		0.981	
Oscillamide Y				0.978		0.996	
Oscillapeptin J				0.951		0.991	

Table S11. Structure of anabaenopeptins. The structure of anabaenopeptin B is shown as an example and the moieties that change for other variants of the same class are highlighted and numbered. The table below provides the changing moieties for all the anabaenopeptins. Modifications indicated in bold are expected to induce a change in reactivity compared to anabaenopeptin B. The circle indicates the expected main attack sites of O_3.

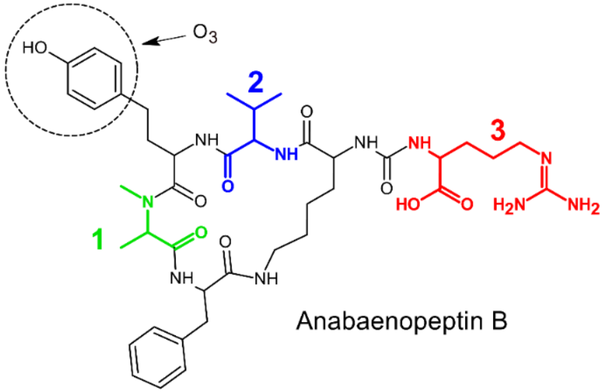

Molecule Name	**1**	**2**	**3**
Anabaenopeptin B	*N*-methylalanine	Valine	Arginine
Anabaenopeptin A	*N*-methylalanine	Valine	**Tyrosine**
Anabaenopeptin F	*N*-methylalanine	Isoleucine	Arginine
Oscillamide Y	*N*-methylalanine	Isoleucine	**Tyrosine**
Anabaenopeptin SA13	*N*-methylserine	Valine	**Tyrosine**

Table S12. Structure of cyanopeptolins. The structure of cyanopeptolin D is shown as an example and the moieties that change for other variants of the same class are highlighted and numbered. The table below provides the changing moieties for all the cyanopeptolins. Modifications indicated in bold are expected to induce a change in reactivity compared to cyanopeptolin D. The circle indicates the expected main attack sites of O_3.

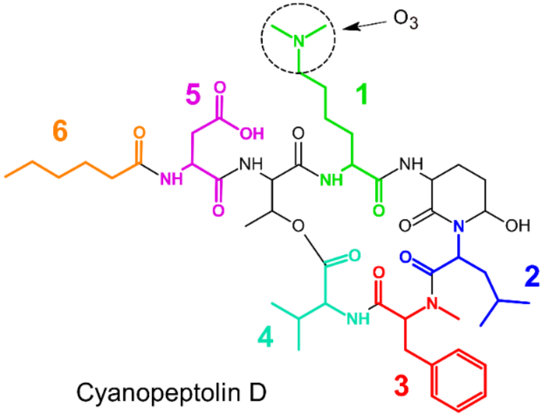

Molecule Name	1	2	3	4	5	6
Cyanopeptolin D	(6-*N*,6-*N*)dimethyl-lysine	Leucine	N-methylphenylalanine	Valine	Aspartic acid	Hexanoic acid
Cyanopeptolin A	**Arginine**	Leucine	N-methylphenylalanine	Valine	Aspartic acid	Hexanoic acid
Cyanopeptolin B	**Lysine**	Leucine	N-methylphenylalanine	Valine	Aspartic acid	Hexanoic acid
Cyanopeptolin C	**6-*N*-methyl-lysine**	Leucine	N-methylphenylalanine	Valine	Aspartic acid	Hexanoic acid
Cyanopeptolin 963A	**Tyrosine**	Leucine	N-methylphenylalanine	Valine	Aspartic acid	Hexanoic acid
Cyanopeptolin 1020	**Arginine**	Phenylalanine	***N*-methyltyrosine**	Valine	Glutamic acid	Hexanoic acid
Oscillapeptin J	**Arginine**	Threonine	***N*-methyltyrosine**	Valine	**Tyrosine**	2-Hydroxy-3-sulfooxypropanoic acid

Table S13. Structure of cyclamides. The structure of aerucyclamide C is shown as an example and the moieties that change for other variants of the same class are highlighted and numbered. The table below provides the changing moieties for all the cyclamides. Modifications indicated in bold are expected to induce a change in reactivity compared to aerucyclamide C. The circle indicates the expected main attack sites of O_3.

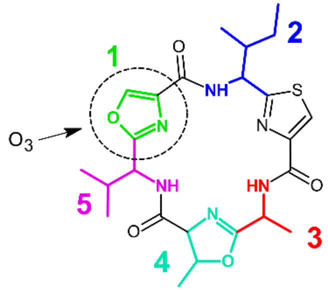

Aerucyclamide C

Molecule Name	1	2	3	4	5
Aerucyclamide C	Oxazole	Isoleucine	Alanine	Methyloxazoline	Valine
Aerucyclamide A	**Methyloxazoline**	Glycine	Isoleucine	Thiazoline	Isoleucine
Aerucyclamide B	**Methyloxazoline**	Glycine	Isoleucine	Thiazole	Isoleucine
Aerucyclamide D	**Methyloxazoline**	Glycine	**Methionine**	Thiazoline	Phenylalanine
Microcyclamide 7806A	Oxazole	Isoleucine	Alanine	O-Threonine	Valine
Microcyclamide 7806B	Oxazole	Isoleucine	Alanine	Threonine	Valine

Table S14. Structure of microcystins. The structure of MC-LR is shown as an example and the moieties that change for other variants of the same class are highlighted and numbered. The table below provides the changing moieties for all the microcystins. Modifications indicated in bold are expected to induce a change in reactivity compared to MC-LR. Circles indicate the expected main attack sites of O_3.

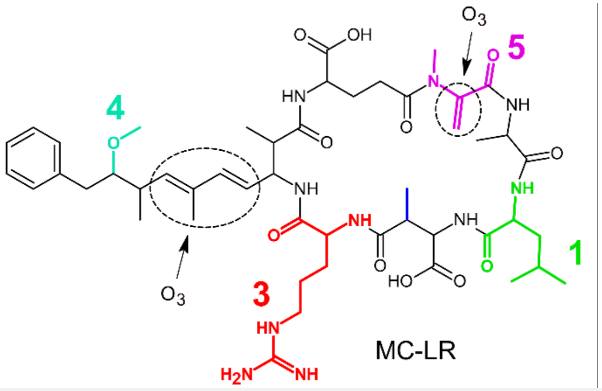

Molecule Name	1	2	3	4	5
MC-LR	Leucine	$-CH_3$	Arginine	$-OCH_3$	*N*-methyldehydroalanine
[Mdha-GSH7]MC-LR[a]	Leucine	$-CH_3$	Arginine	$-OCH_3$	***N*-methyldehydroalanine-Glutathione**
MC-LAba	Leucine	$-CH_3$	2-aminobutanoic acid	$-OCH_3$	*N*-methyldehydroalanine
[D-Asp3]MC-LA	Leucine	-H	Alanine	$-OCH_3$	*N*-methyldehydroalanine
[Asp3,DMAdda5,Dhb7]MC-LR or [D-Asp3,DMAdda5]MC-LR	Leucine	-H	Arginine	-OH	2-(methylamino)but-2-enoic acid or *N*-methyldehydroalanine
[D-Asp3]MC-LR	Leucine	-H	Arginine	$-OCH_3$	*N*-methyldehydroalanine
MC-HilR	Homoisoleucine	$-CH_3$	Arginine	$-OCH_3$	*N*-methyldehydroalanine
[D-Asp3,(E)-Dhb7]MC-RR	Arginine	-H	Arginine	$-OCH_3$	2-aminobut-2-enoic acid
[D-Asp3,Dha7]MC-RR	Arginine	-H	Arginine	$-OCH_3$	Dehydroalanine
[D-Asp3,DMAdda5]MC-RR	Arginine	-H	Arginine	-OH	*N*-methyldehydroalanine

[a]The full structure is shown in Figure S5

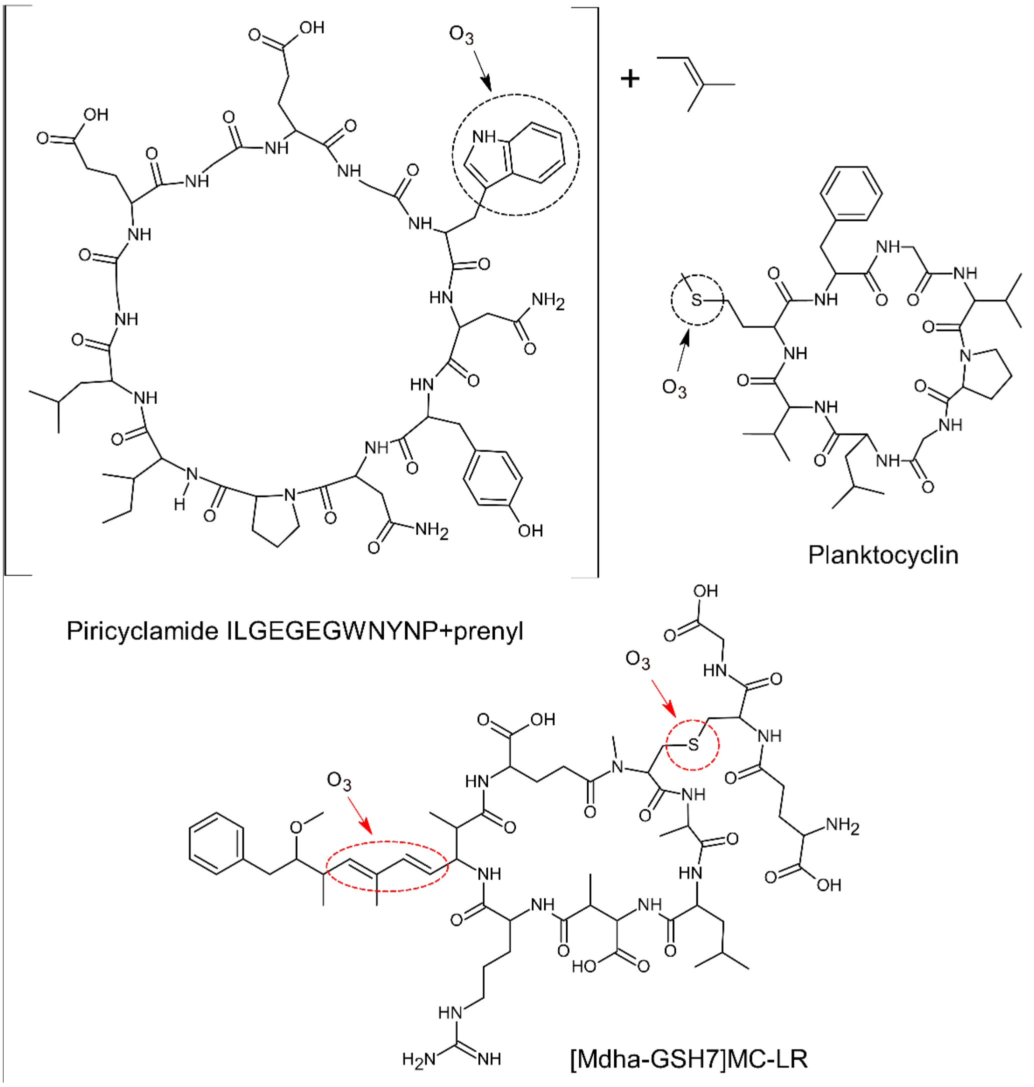

Figure S5. Structure of two unclassified cyano-metabolites, piricyclamide ILGEGEGWNYNP and planktocyclin, and of the microcystin [Mdha-GSH7]MC-LR. Circles indicate the primary attack sites of O_3. For [Mdha-GSH7]MC-LR, it is undefined which of the thioether or olefin is the primary attack site (see explanation in the main manuscript). The exact prenylation site on piricyclamide ILGEGEGWNYNP is unknown.

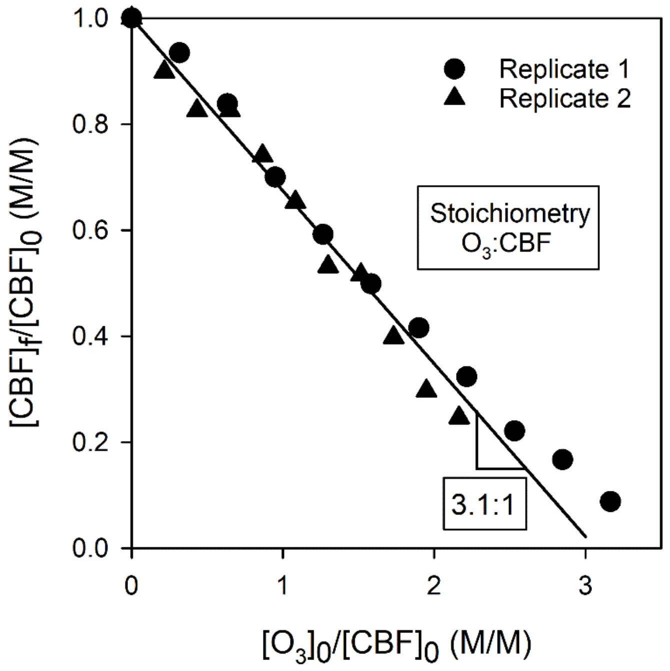

Figure S6. Stoichiometry for the reaction between O_3 and carbofuran (CBF). Replicates 1 and 2 were conducted on separate days, using a freshly prepared CBF stock solution. $[CBF]_0$ = 133-266 µM, $[O_3]_0$ = 40-400 µM. pH was adjusted to about 3-4, similar to the pH used by David Yao and Haag,[8] with phosphate/phosphoric acid (10 mM).

Text S4. Determination of k_{DMP}

The second-order rate constant for the reaction of dibromomethylparaben (DMP) with O_3 was re-determined at pH 6, 7 and 8 using cinnamic acid as a competitor ($k_{O3,cinnamic\ acid} = 5.8 \times 10^4$ M^{-1} s^{-1}, $k_{O3,cinnamate}$ $(7.5 \pm 0.4) \times 10^5$ M^{-1} s^{-1}, pK_a = 4.4)[14] and at pH 7 using phenol as a competitor ($k_{O3,phenol} = (1.3 \pm 0.2) \times 10^3$ M^{-1} s^{-1}, $k_{O3,phenolate}$ $(1.4 \pm 0.4) \times 10^9$ M^{-1} s^{-1}, pK_a = 9.9).[19] The experiments were performed with 3 µM of DMP and 3 µM of cinnamic acid or phenol and O_3 doses of 0.4-4 µM, at 24±1°C in presence of 2 mM phosphate and 40 mM *tert*-butanol. The corresponding results are shown in Table S15. Less than 10% variation was observed between $k_{app,O3}$ determined with cinnamic acid between pH 6 and 8, consistent with the low pK_a of DMP (4.7).[11] A $k_{O3,DMP}$ of $(4.3 \pm 0.3) \times 10^6$ M^{-1} s^{-1} was calculated for the deprotonated form by averaging the k_{app} at pH 7 and 8 (for which >99% of DMP is deprotonated). The $k_{app,O3,DMP}$ determined by phenol at pH 7 was within 5% of the $k_{app,O3,DMP}$ determined by cinnamic acid, further validating our determination. The higher uncertainty on the $k_{app,O3}$ determined with phenol (± 1.2×10^6 M^{-1} s^{-1}) compared to the $k_{app,O3}$ determined with cinnamic acid (± 0.2-0.3 × 10^6 M^{-1} s^{-1}) is due to the higher uncertainty on the $k_{O3,phenolate}$ compared to $k_{O3,cinnamate}$.[14, 19]

Table S15. $k_{app,O3}$ of dibromomethylparaben (DMP) determined by cinnamic acid and phenol at 24±1°C. Each experiment was performed in triplicate.

Competitor	pH	Number of data points	R^2	p-value	$k_{app,O3}$
Cinnamic acid	5.97±0.03	19	0.973	<0.0001	$(4.0 \pm 0.3) \times 10^6$
Cinnamic acid	6.94±0.01	22	0.977	<0.0001	$(4.3 \pm 0.3) \times 10^6$
Cinnamic acid	7.97±0.02	22	0.996	<0.0001	$(4.4 \pm 0.2) \times 10^6$
Phenol	6.97±0.02	19	0.991	<0.0001	$(4.4 \pm 1.2) \times 10^6$

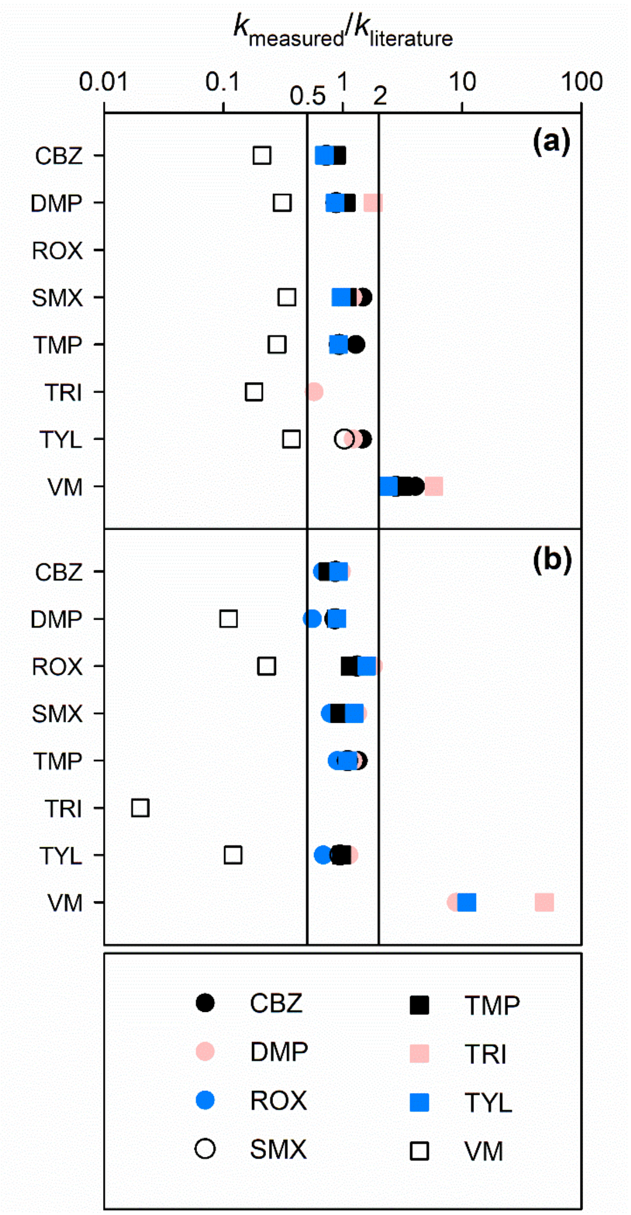

Figure S7. Adjusted $k_{measured}/k_{literature}$ of competitors in the presence of the cyano-metabolite mixture from 0.4 $g_{biomass}$ L^{-1} of *Planktothrix* at (a) pH 7 and (b) pH 8 (2 mM phosphate), 22°C and in presence of *tert*-butanol (40 mM). The vertical lines correspond to the limits for acceptable $k_{measured}/k_{literature}$ range, set between 0.5 and 2.

Table S16. Apparent second-order rate constants (k_{app}, $M^{-1}s^{-1}$) at pH 7 for the reactions of ozone with cyano-metabolites from *Planktothrix* and *Microcystis*. The results for all obtained k_{app} (including standard errors (SE)) for the corresponding competitors are shown.

**Cyano-metabolites** / **Competitors**		**TMP**	**CBZ**	**TYL**	**SMX**	**DMP**	**TRI**	**Averaged k_{app}**	**SE**
**Tryptophan**									
Piricyclamide ILGEGEGWNYNP+prenyl	k_{app}						7.3×10^7	7.3×10^7	1.7×10^7
	SE						1.7×10^7		
**Thioethers**									
[Mdha-GSH7]MC-LR	k_{app}	2.0×10^6		1.5×10^6	1.9×10^6	2.2×10^6		1.9×10^6	4.3×10^5
	SE	4.3×10^5		2.9×10^5	3.6×10^5	1.2×10^5			
Aerucyclamide D	k_{app}					4.1×10^7	6.2×10^7	5.2×10^7	1.5×10^7
	SE					4.9×10^6	1.4×10^7		
Planktocyclin	k_{app}					2.8×10^7	4.0×10^7	3.4×10^7	9×10^6
	SE					4.0×10^6	9.0×10^6		
**Olefins**									
[D-Asp3,(E)-Dhb7]MC-RR	k_{app}	1.6×10^6	2.2×10^6	1.3×10^6	1.4×10^6	1.8×10^6		1.7×10^6	3.3×10^5
	SE	3.3×10^5	1.7×10^5	2.9×10^5	3.0×10^5	1.1×10^5			
[D-Asp3,Dha7]MC-RR	k_{app}	1.2×10^6	1.5×10^6	1.1×10^6	1.1×10^6	1.5×10^6		1.3×10^6	2.5×10^5
	SE	2.5×10^5	1.3×10^5	2.0×10^5	2.1×10^5	1.3×10^5			
[D-Asp3,DMAdda5]MC-RR	k_{app}	1.6×10^6	2.2×10^6	1.3×10^6	1.5×10^6	1.9×10^6		1.7×10^6	3.4×10^5
	SE	3.4×10^5	1.8×10^5	2.5×10^5	2.6×10^5	1.3×10^5			
[D-Asp3]MC-LA	k_{app}	1.0×10^6	1.3×10^6	8.6×10^5	8.9×10^5			9.7×10^5	2.1×10^5
	SE	2.1×10^5	6.9×10^4	1.6×10^5	1.6×10^5				
[D-Asp3]MC-LR (Planktothrix)	k_{app}	1.1×10^6	1.4×10^6	8.8×10^5	9.8×10^5	1.2×10^6		1.1×10^6	2.1×10^5
	SE	2.1×10^5	5.7×10^4	1.6×10^5	1.7×10^5	1.2×10^5			
[D-Asp3]MC-LR (Microcystis)	k_{app}	1.1×10^6	1.3×10^6	8.9×10^5	9.4×10^5			1.1×10^6	2.1×10^5
	SE	2.1×10^5	6.0×10^4	1.7×10^5	1.7×10^5				
MC-HilR	k_{app}	1.1×10^6	1.5×10^6	9.2×10^5	1.0×10^6	1.1×10^6		1.1×10^6	2.3×10^5
	SE	2.3×10^5	7.4×10^4	1.7×10^5	1.9×10^5	1.5×10^5			

Table S16 continued.

MC-LAba	k_{app}	1.1×10^6	1.4×10^6	9.5×10^5	1.0×10^6	1.2×10^6	1.1×10^6	2.2×10^5
	SE	2.2×10^5	9.8×10^4	1.8×10^5	1.8×10^5	1.2×10^5		
MC-LR (Planktothrix)	k_{app}	1.1×10^6	1.4×10^6	8.8×10^5	9.8×10^5	1.2×10^6	1.1×10^6	2.1×10^5
	SE	2.1×10^5	5.6×10^4	1.6×10^5	1.8×10^5	1.1×10^5		
MC-LR (Microcystis)	k_{app}	1.0×10^6	1.3×10^6	8.9×10^5	9.4×10^5	1.2×10^6	1.1×10^6	2.1×10^5
	SE	2.1×10^5	6.5×10^4	1.7×10^5	1.7×10^5	5.5×10^4		
Microcystin-group-967	k_{app}	1.0×10^6	1.3×10^6	8.6×10^5	9.3×10^5	1.3×10^6	1.1×10^6	2.1×10^5
	SE	2.1×10^5	8.0×10^4	1.6×10^5	1.7×10^5	1.4×10^5		
**Phenols**								
Anabaenopeptin A	k_{app}	1.1×10^6	1.5×10^6	1.0×10^6	1.1×10^6	1.3×10^6	1.2×10^6	2.3×10^5
	SE	2.3×10^5	6.0×10^4	1.9×10^5	1.9×10^5	3.1×10^4		
Anabaenopeptin B	k_{app}	9.4×10^5	1.2×10^6	8.4×10^5	8.8×10^5		9.7×10^5	1.9×10^5
	SE	1.9×10^5	6.3×10^4	1.6×10^5	1.6×10^5			
Anabaenopeptin F	k_{app}	1.0×10^6	1.3×10^6	8.8×10^5	9.2×10^5		1.0×10^6	2.1×10^5
	SE	2.1×10^5	1.0×10^5	1.7×10^5	1.7×10^5			
Anabaenopeptin SA13	k_{app}	1.2×10^6	1.5×10^6	1.0×10^6	1.1×10^6	1.4×10^6	1.2×10^6	2.4×10^5
	SE	2.4×10^5	1.1×10^5	2.0×10^5	1.9×10^5	1.0×10^5		
Aeruginosin-group-608	k_{app}	1.5×10^6	2.0×10^6	1.2×10^6	1.4×10^6	1.7×10^6	1.6×10^6	3.2×10^5
	SE	3.2×10^5	1.7×10^5	2.3×10^5	2.4×10^5	1.2×10^5		
Cyanopeptolin 1020	k_{app}	5.7×10^5	7.3×10^5	4.8×10^5	5.0×10^5		5.7×10^5	1.2×10^5
	SE	1.2×10^5	2.8×10^4	9.2×10^4	9.1×10^4			
Cyanopeptolin 963A	k_{app}	3.6×10^5	4.7×10^5	2.7×10^5	3.2×10^5		3.5×10^5	7.9×10^4
	SE	7.6×10^4	3.2×10^4	5.7×10^4	6.7×10^4			
Oscillamide Y	k_{app}	1.2×10^6	1.5×10^6	1.0×10^6	1.1×10^6		1.3×10^6	2.5×10^5
	SE	2.5×10^5	1.2×10^5	1.9×10^5	1.9×10^5			
Oscillapeptin J	k_{app}	1.6×10^6	2.0×10^6	1.3×10^6	1.5×10^6	1.8×10^6	1.6×10^6	3.7×10^5
	SE	3.7×10^5	1.7×10^5	2.6×10^5	2.7×10^5	1.7×10^5		

Table S16 continued.

Cyano-metabolites \ Competitors		ASMX	BZF	CBF	TRA	PG	CIP	Averaged k_{app}	SE
**Tertiary amine**									
Cyanopeptolin C	k_{app}	9.4×10^1	1.2×10^2	9.9×10^1				1.0×10^2	2.9×10^1
	SE	0.8×10^1	1.6×10^1	2.9×10^1					
Cyanopeptolin D	k_{app}	1.9×10^3	2.3×10^3	1.6×10^3	1.5×10^3	2.7×10^3		2.0×10^3	4.9×10^2
	SE	1.9×10^2	2.3×10^2	4.9×10^2	3.5×10^2	1.2×10^2			
**Heterocycles**									
Aerucyclamide A	k_{app}	8.3×10^1	1.1×10^2	7.7×10^1				8.8×10^1	1.6×10^1
	SE	0.5×10^1	1.6×10^1	0.8×10^1					
Aerucyclamide C	k_{app}		4.5×10^3		3.0×10^3	5.0×10^3		4.2×10^3	1.1×10^3
	SE		5.2×10^2		6.9×10^2	1.6×10^2			
Microcyclamide 7806A	k_{app}		5.0×10^3		3.3×10^3	5.7×10^3		4.7×10^3	1.2×10^3
	SE		7.1×10^2		7.6×10^2	1.8×10^2			
Microcyclamide 7806B	k_{app}		5.3×10^3		3.7×10^3	6.1×10^3		5.0×10^3	1.1×10^3
	SE		7.0×10^2		8.4×10^2	2.5×10^2			

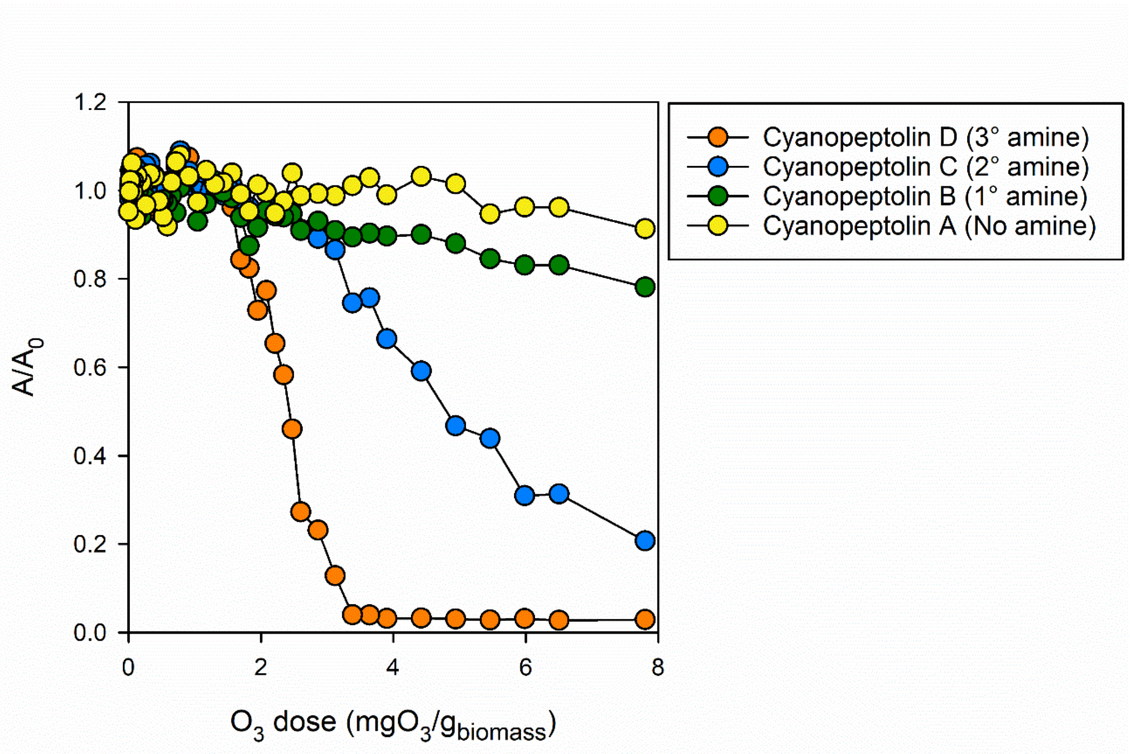

Figure S8. Abatement of cyanopeptolin D, C, B and A from 0.6 $g_{biomass}$ L^{-1} of *Microcystis* at pH 7 as a function of the O_3 dose at pH 7 (2 mM phosphate), 22°C, and in presence of *tert*-butanol (40 mM).

Table S17. pK_a of amine-containing compounds

	Predicted by ChemAxon	CRC handbook (95th edition)[20]
Ethylamine (1° amine)	10.23	10.65
Diethylamine (2° amine)	11.18	10.84
Triethylamine (3° amine)	10.81	10.75
Cyanopeptolin B (1° amine)	10.18	
Cyanopeptolin C (2° amine)	11.58	
Cyanopeptolin D (3° amine)	10.35	

Text S5. Estimation of cyanopeptolin B and C formation during ozonation experiments

Cyanopeptolin B, cyanopeptolin C and cyanopeptolin D have the same structure, apart from the amine-containing moieties (Table S12). Cyanopeptolin B contains a lysine in which the primary amine of the side chain is the reaction site. Cyanopeptolins C and D contain lysine derivatives, in which the side chain amine is substituted by one and two methyl groups, respectively. Upon ozonation, it has been shown that dealkylation of an amine can occur, i.e., a tertiary amine is oxidized to the corresponding secondary amine and a secondary amine is oxidized to the corresponding primary amine.[21] That is, cyanopeptolin C (secondary amine) can be formed from cyanopeptolin D (tertiary amine) ozonation and cyanopeptolin B (primary amine) can be formed from cyanopeptolin C (secondary amine) ozonation. For triethylamine and diethylamine, dealkylation yields are 5 and 8%, respectively.[21] To estimate if the formation of cyanopeptolin B (primary amine) and C (secondary amine) was significant compared to their initial concentration, it is important to consider the relative initial concentrations of the three cyanopeptolins. The relative concentrations of these three cyanopeptolins can only be estimated based on peak areas as no standards are available for cyanopeptolin B and C. Based on peak areas of both $[M+H]^+$ and $[M-H]^-$ ions, the initial cyanopeptolin C (secondary amine) concentration was about half of that of cyanopeptolin D (tertiary amine), and the initial cyanopeptolin B (primary amine) concentration was about half of that of cyanopeptolin C (secondary amine). Combining these estimations with previously determined dealkylation yields, up to about 10% of the initial cyanopeptolin C (secondary amine) might have been formed from cyanopeptolin D (tertiary amine) ozonation, and hence the $k_{app,O3}$ of cyanopeptolin C may be underestimated by up to about 10%. Similarly, up to about 16% of the initial cyanopeptolin B (primary amine) might have been formed from cyanopeptolin C (secondary amine) ozonation. These estimations consider that none of the dealkylations involve the detachment of the amine group from the cyanopeptolin, which may happen and would not lead to a known cyanopeptolin, reducing the estimated yields.

Table S18. Apparent second-order rate constants (k_{app}, $M^{-1}s^{-1}$) at pH 8 for the reactions of ozone with cyano-metabolites from *Planktothrix*. The results for all obtained k_{app} (including standard errors (SE)) for the corresponding competitors are shown.

Competitors / Cyano-metabolites		ROX	TMP	CBZ	TYL	SMX	DMP	TRI	Averaged *k*app	SE
**Tryptophan**										
Piricyclamide	k_{app}						3.1×10^{7}	1.9×10^{8}	N/A	N/A
ILGEGEGWNYNP+prenyl	SE						3.5×10^{6}	2.3×10^{7}		
Thioether										
Planktocyclin	k_{app}						1.8×10^{7}	1.1×10^{8}	N/A	N/A
	SE						1.9×10^{6}	1.7×10^{7}		
**Olefins**										
[D-Asp3]MC-LR	k_{app}	8.6×10^{5}	9.0×10^{5}	1.2×10^{6}		8.9×10^{5}	8.3×10^{5}		9.5×10^{5}	1.4×10^{5}
	SE	1.8×10^{5}	1.7×10^{5}	5.2×10^{4}		1.6×10^{5}	1.2×10^{5}			
[D-Asp3,(E)-Dhb7]MC-RR	k_{app}	1.2×10^{6}	1.4×10^{6}	1.7×10^{6}	1.5×10^{6}	1.4×10^{6}	1.6×10^{6}		1.5×10^{6}	1.8×10^{5}
	SE	2.4×10^{5}	2.7×10^{5}	1.2×10^{5}	2.9×10^{5}	2.5×10^{5}	1.2×10^{5}			
[D-Asp3]MC-LA	k_{app}	8.0×10^{5}	8.5×10^{5}	1.1×10^{6}		9.0×10^{5}	1.0×10^{6}		9.1×10^{5}	1.9×10^{5}
	SE	1.6×10^{5}	1.6×10^{5}	4.9×10^{4}		1.6×10^{5}	9.7×10^{4}			
[D-Asp3,Dha7]MC-RR	k_{app}	1.0×10^{6}	1.1×10^{6}	1.4×10^{6}	1.3×10^{6}	1.2×10^{6}	1.4×10^{6}		1.2×10^{6}	1.5×10^{5}
	SE	2.0×10^{5}	2.1×10^{5}	8.4×10^{4}	2.5×10^{5}	2.1×10^{5}	1.4×10^{5}			
[D-Asp3,DMAdda5]MC-RR	k_{app}	1.2×10^{6}	1.4×10^{6}	1.7×10^{6}	1.6×10^{6}	1.4×10^{6}	1.8×10^{6}		1.5×10^{6}	2.3×10^{5}
	SE	2.6×10^{5}	2.8×10^{5}	1.2×10^{5}	3.0×10^{5}	2.6×10^{5}	1.4×10^{5}			
MC-LR	k_{app}	9.0×10^{5}	9.3×10^{5}	1.2×10^{6}		9.8×10^{5}	1.0×10^{6}		1.0×10^{6}	1.2×10^{5}
	SE	1.8×10^{5}	1.8×10^{5}	4.7×10^{4}		1.8×10^{5}	8.8×10^{4}			
MC-LAba	k_{app}	9.5×10^{5}	9.8×10^{5}	1.3×10^{6}	1.2×10^{6}	1.0×10^{6}	1.1×10^{6}		1.1×10^{6}	1.3×10^{5}
	SE	1.9×10^{5}	1.9×10^{5}	4.5×10^{4}	2.2×10^{5}	1.8×10^{5}	1.4×10^{5}			
**Phenols**										
Anabaenopeptin A	k_{app}				7.9×10^{6}		7.5×10^{6}		7.7×10^{6}	1.6×10^{6}
	SE				1.6×10^{6}		5.5×10^{5}			

Table S18 continued.

Anabaenopeptin B	k_{app}		6.0×10^6	5.8×10^6	5.9×10^6	1.2×10^6
	SE		1.2×10^6	4.3×10^5		
Anabaenopeptin F	k_{app}		6.9×10^6	6.5×10^6	6.7×10^6	1.5×10^6
	SE		1.3×10^6	5.7×10^5		
Anabaenopeptin SA13	k_{app}		9.0×10^6	8.6×10^6	8.8×10^6	1.7×10^6
	SE		1.7×10^6	7.1×10^5		
Cyanopeptolin 1020	k_{app}		4.1×10^6	4.2×10^6	4.1×10^6	7.5×10^5
	SE		7.5×10^5	3.0×10^5		
Aeruginosin-group-608	k_{app}		8.9×10^6	8.5×10^6	8.7×10^6	1.5×10^6
	SE		1.8×10^6	7.3×10^5		
Oscillamide Y	k_{app}		8.8×10^6	8.1×10^6	8.4×10^6	1.7×10^6
	SE		1.7×10^6	6.1×10^5		
Oscillapeptin J	k_{app}		1.1×10^7	1.1×10^7	1.1×10^7	2.1×10^6
	SE		2.1×10^6	8.8×10^5		

REFERENCES

1. Guillard, R. R. L.; Lorenzen, C. J., Yellow-green algae with chlorophyllide C1,2. *J. Phycol.* **1972,** *8*, (1), 10-14.
2. Natumi, R.; Marcotullio, S.; Janssen, E. M. L., Phototransformation kinetics of cyanobacterial toxins and secondary metabolites in surface waters. *Environ. Sci. Eur.* **2021,** *33*, (1), 26.
3. Natumi, R.; Dieziger, C.; Janssen, E. M. L., Cyanobacterial Toxins and Cyanopeptide Transformation Kinetics by Singlet Oxygen and pH-Dependence in Sunlit Surface Waters. *Environ. Sci. Technol.* **2021,** *55*, (22), 15196-15205.
4. Jones, M.; Janssen, E. M. L., Quantification of Multi-class Cyanopeptides in Swiss Lakes with Automated Extraction, Enrichment and Analysis by Online-SPE HPLC-HRMS/MS. *CHIMIA* **2022,** *76*, (1-2), 133-144.
5. Ruttkies, C.; Schymanski, E. L.; Wolf, S.; Hollender, J.; Neumann, S., MetFrag relaunched: incorporating strategies beyond in silico fragmentation. *J. Cheminformatics* **2016,** *8*, (1), 3.
6. Maleknia, S. D.; Johnson, R., Mass Spectrometry of Amino Acids and Proteins. In *Amino Acids, Peptides and Proteins in Organic Chemistry*, 2011; pp 1-50.
7. Dodd, M. C.; Buffle, M.-O.; von Gunten, U., Oxidation of Antibacterial Molecules by Aqueous Ozone: Moiety-Specific Reaction Kinetics and Application to Ozone-Based Wastewater Treatment. *Environ. Sci. Technol.* **2006,** *40*, (6), 1969-1977.
8. David Yao, C. C.; Haag, W. R., Rate constants for direct reactions of ozone with several drinking water contaminants. *Water Res.* **1991,** *25*, (7), 761-773.
9. Huber, M. M.; Canonica, S.; Park, G.-Y.; von Gunten, U., Oxidation of Pharmaceuticals during Ozonation and Advanced Oxidation Processes. *Environ. Sci. Technol.* **2003,** *37*, (5), 1016-1024.
10. Wolf, C.; von Gunten, U.; Kohn, T., Kinetics of Inactivation of Waterborne Enteric Viruses by Ozone. *Environ. Sci. Technol.* **2018,** *52*, (4), 2170-2177.
11. Lee, W.; Marcotullio, S.; Yeom, H.; Son, H.; Kim, T.-H.; Lee, Y., Reaction kinetics and degradation efficiency of halogenated methylparabens during ozonation and UV/H2O2 treatment of drinking water and wastewater effluent. *J. Hazard. Mater.* **2022,** *427*, 127878.
12. Zimmermann, S. G.; Schmukat, A.; Schulz, M.; Benner, J.; Gunten, U. v.; Ternes, T. A., Kinetic and Mechanistic Investigations of the Oxidation of Tramadol by Ferrate and Ozone. *Environ. Sci. Technol.* **2012,** *46*, (2), 876-884.
13. Suarez, S.; Dodd, M. C.; Omil, F.; von Gunten, U., Kinetics of triclosan oxidation by aqueous ozone and consequent loss of antibacterial activity: Relevance to municipal wastewater ozonation. *Water Res.* **2007,** *41*, (12), 2481-2490.
14. Kim, M. S.; Lee, C., Ozonation of Microcystins: Kinetics and Toxicity Decrease. *Environ. Sci. Technol.* **2019,** *53*, (11), 6427-6435.
15. Schymanski, E. L.; Jeon, J.; Gulde, R.; Fenner, K.; Ruff, M.; Singer, H. P.; Hollender, J., Identifying Small Molecules via High Resolution Mass Spectrometry: Communicating Confidence. *Environ. Sci. Technol.* **2014,** *48*, (4), 2097-2098.
16. Portmann, C.; Blom, J. F.; Kaiser, M.; Brun, R.; Jüttner, F.; Gademann, K., Isolation of Aerucyclamides C and D and Structure Revision of Microcyclamide 7806A: Heterocyclic Ribosomal Peptides from Microcystis aeruginosa PCC 7806 and Their Antiparasite Evaluation. *J. Nat. Prod.* **2008,** *71*, (11), 1891-1896.
17. Portmann, C.; Blom, J. F.; Gademann, K.; Jüttner, F., Aerucyclamides A and B: Isolation and Synthesis of Toxic Ribosomal Heterocyclic Peptides from the Cyanobacterium Microcystis aeruginosa PCC 7806. *J. Nat. Prod.* **2008,** *71*, (7), 1193-1196.

18. Ziemert, N.; Ishida, K.; Quillardet, P.; Bouchier, C.; Hertweck, C.; de Marsac Nicole, T.; Dittmann, E., Microcyclamide Biosynthesis in Two Strains of Microcystis aeruginosa: from Structure to Genes and Vice Versa. *Applied and Environmental Microbiology* **2008,** *74,* (6), 1791-1797.
19. Hoigné, J.; Bader, H., Rate constants of reactions of ozone with organic and inorganic compounds in water—II. *Water Res.* **1983,** *17*, (2), 185-194.
20. Haynes, W. M., *CRC Handbook of Chemistry and Physics (95th Edition)*. 95th ed.; CRC Press: Hoboken, 2014.
21. Lim, S.; McArdell, C. S.; von Gunten, U., Reactions of aliphatic amines with ozone: Kinetics and mechanisms. *Water Res.* **2019,** *157*, 514-528.
